# Association Mapping for Important Agronomic Traits in Core Collection of Rice (*Oryza sativa* L.) with SSR Markers

**DOI:** 10.1371/journal.pone.0111508

**Published:** 2014-10-31

**Authors:** Peng Zhang, Xiangdong Liu, Hanhua Tong, Yonggen Lu, Jinquan Li

**Affiliations:** 1 State Key Laboratory for Conservation and Utilization of Subtropical Agro-bioresources, South China Agricultural University, Guangzhou, China; 2 State Key Laboratory of Rice Biology, China National Rice Research Institute, Hangzhou, China; 3 Department of Plant Breeding and Genetics, Max Planck Institute for Plant Breeding Research, Cologne, Germany; International Rice Research Institute, Philippines

## Abstract

Mining elite genes within rice landraces is of importance for the improvement of cultivated rice. An association mapping for 12 agronomic traits was carried out using a core collection of rice consisting of 150 landraces (Panel 1) with 274 simple sequence repeat (SSR) markers, and the mapping results were further verified using a Chinese national rice micro-core collection (Panel 2) and a collection from a global molecular breeding program (Panel 3). Our results showed that (1) 76 significant (*P*<0.05) trait-marker associations were detected using mixed linear model (MLM) within Panel 1 in two years, among which 32% were identical with previously mapped QTLs, and 11 significant associations had >10% explained ratio of genetic variation; (2) A total of seven aforementioned trait-marker associations were verified within Panel 2 and 3 when using a general linear model (GLM) and 55 SSR markers of the 76 significant trait-marker associations. However, no significant trait-marker association was found to be identical within three panels when using the MLM model; (3) several desirable alleles of the loci which showed significant trait-marker associations were identified. The research provided important information for further mining these elite genes within rice landraces and using them for rice breeding.

## Introduction

As a staple cereal crop, rice (*Oryza sativa* L.) feeds more than 50% of the world's population [1] and is one of the most important components of human diet in many regions of the world. Thus, genetic improvement of rice for yield is important to the meet food demand of a growing global population. Rice landraces have a greater genetic diversity than elite cultivars (or commercial cultivars) and represent an intermediate stage in domestication between wild rice and elite cultivars [2], which make it easier to be used in rice breeding than wild rice and at the same time still keeping most of the diversity in rice germplasm resource. Therefore, mining elite genes within the germplasm of rice landraces is of importance for the improvement of cultivated rice.

Linkage mapping and association mapping based linkage disequilibrium (LD) are two main methods for locating genes or QTLs. The major limitations of linkage mapping are that only two alleles at any given locus can be studied in bi-parental crosses and a low mapping resolution [3], whereas association mapping promises to overcome the limitations of linkage mapping [4]. Moreover, association mapping identifies QTLs by examining the trait-marker associations and enables researchers to use modern genetic technologies to exploit natural diversity and locate valuable genes in the genome [5].

Association mapping has been widely used in plant research since it was firstly reported in maize [6], [7]. In recent years, association mapping has been applied in *Arabidopsis*, maize, barley, durum wheat, spring wheat, sorghum, sugarcane, sugar beet, soybean, grape, forest tree species and forage grasses [8] as well as rice [9], [10], [11], [12]. For example, an association mapping was performed with 60 simple sequence repeat (SSR) markers and 114 restriction fragment length polymorphism (RFLP) markers for 12 agronomic traits within 218 inbred lines of rice originating from United States of America (USA) and Asia [13]. An association mapping was performed for five agronomic traits in a population of 103 cultivars using 123 SSRs [14] as well as for grain shape using a collection of 293 accessions of Asian cultivated rice [15]. An association mapping for starch quality traits using both candidate gene-based association mapping and genome-wide association study (GWAS) strategies was performed [16]. More than 3.6 million SNPs were detected by sequencing 517 rice landraces and applied for GWAS for 14 agronomic traits [17]. However, to our knowledge, an association mapping with a high number of SSR markers was seldom performed in the previous studies. Moreover, no earlier research performed an association mapping in one population and at the same time verified the association mapping results in other populations.

The choice of appropriate germplasm to maximize the number of historical recombinations and mutation events (and thus reduce LD) within and around the gene of interest is critical for the success of association analysis [18]. One of the methods to obtain most of the phenotypes is to construct a core collection. A core collection is a subset chosen to represent most genetic diversity of an initial collection with a minimum of redundancies [19], [20], [21]. Core collections facilitate the users to access useful samples of small sizes while still keeping most of the genetic variability contained within the gene pool of a specific crop [22]. The construction of a core collection was widely applied in rice as well as other crops. Thus, a core collection might be an ideal mapping population for association mapping. Some rice core collections have been used as association mapping populations in previous studies [23], [24]. However, the mapping population in the studies mentioned above were two subsets consisting of 547 and 203 accessions chosen randomly from United States Department of Agriculture (USDA) rice core collection which consists of 1790 rice entries, which cannot effectively maintain the genetic diversity in the original collections. Moreover, the number of SSR markers for genotyping was low (72 and 155) in the studies. As far as we know, no earlier research on association mapping based on a core collection of rice landraces was available.

Population structure may cause false positives in association mapping. To overcome this problem, an approach using a mixed-model was proposed for association mapping, which take both population structure (Q) and kinship (K) into account for the reduction of false positives [25]. In recent years, comparisons of different statistical models e.g. Q, Q+K and P+K have been conducted for *Arabidopsis* [26], sweet sorghum [27], maize [28] and rice [23]. However, false positive might not be absolutely avoided through the aforementioned models. To avoid them, it required that the significant associations identified within one population should be verified in another population [29].

In our previous studies, a rice core collection (Ting's rice core collection) consisting of 150 accessions of rice landraces has been constructed based on 15 quantitative traits and 34 qualitative traits from 2262 accessions of rice landraces of the Ting's collection with an optimal sampling strategy [30]. Moreover, population structure and LD of the rice core collection had been examined in details [31]. In this study, an association mapping was performed for 12 agronomic traits in the Ting's core collection assessed with 274 SSR markers. Moreover, the significant trait-marker associations identified in the population were verified within a Chinese national rice micro-core collection and a collection from a global molecular breeding program. The study aimed to (1) perform association mapping for 12 important agronomic traits in the Ting's core collection and verify some of the mapping results in another two core collections, (2) compare the effectiveness of different statistical models and different significant thresholds for association mapping, and (3) identify desirable alleles of the loci which showed significant trait-marker associations for rice breeding.

## Materials and Methods

### Plant material

Three rice collections, i.e. Ting's core collection (Panel 1), the Chinese national micro-core collection (Panel 2), and a collection from the core collection of a global molecular breeding program (Panel 3) were used in this study. Panel 1 was collected by the researcher Ying Ting during 1920–1964 from all over China as well as from Korea, Japan, Philippines, Brazil, Celebes, Java, Oceania, and Vietnam. The original collection comprises 7128 rice landraces [32]. The core collection (Panel 1) with 150 accessions was constructed from 2262 accessions of 7128 based on a strategy of stepwise clustering and preferred sampling on adjusted Euclidean distances and weighted pair-group average method using integrated qualitative and quantitative traits [30]. Panel 2 with 197 accessions was provided by China Agricultural University, and Panel 3 with 122 accessions was offered by the International Rice Research Institute (IRRI). The information for each variety is shown in Table S1 in File S1.

### Phenotyping

All of the three panels were cultivated at the farm of South China Agricultural University, Guangzhou (23°16N, 113°8E), during the late season (July-November) for two consecutive years (2008 and 2009). A randomized complete block design with three replications was used during each season. The space between rows and between plants was set to 20 and 16.5 cm, respectively. Thirty plants of each variety were grown in three rows with 10 plants per row. For each block, the five plants in the middle position of the second row of each variety were selected so that the marginal effect was avoided. 12 agronomic traits for these plants were investigated. Heading date (HD) was recorded as days from sowing to flowering time when 30% of the individuals of one variety started flowering. Plant height (PH), panicle length (PL), grain length (GL), grain width (GW), flag leaf length (FLL), and flag leaf width (FLW) were measured in centimeters. Seed set rate (SS, %) was the percentage of filled grains divided by the total grains per plant. For 1000-grain weight (1000GW), 100 grains were measured in grams with three replicates and then its average was multiplied by 10. For grain length (GL) and width (GW), ten grains were randomly selected and measured with a digital vernier caliper.

### Genotyping

274 SSR markers evenly distributed across the 12 chromosomes of rice were selected to genotype all varieties in Panel 1 (Table S2 in File S1). A total of 23, 25, 24, 22, 21, 22, 21, 25, 23, 24, 23, and 21 of these markers were mapped to chromosomes 1 to 12, respectively. The average distance between the loci in chromosomes 1 to 12 is 7.5 cM, 8.2 cM, 9.4 cM, 7.4 cM, 7.1 cM, 6.3 cM, 5.8 cM, 5.4 cM, 5.2 cM, 4.7 cM, 5.6 cM and 5.3 cM, respectively. Markers which prefix RM were summarized in [33], [34], [35], [36] and those with prefix PSM were summarized in [37]. DNA was extracted using a modified SDS method [38]. The volume of the polymerase chain reaction (PCR) was 10 µl. The profile of the PCR program was as follows: 94°C for 5 mins followed by 29 cycles of 94°C for 1 min, 55°C for 1 min, 72°C for 1 min with a final extension of 5 minutes at 72°C. PCR products were separated in size by 6% polyacrylamide gel electrophoresis and detected by silver staining [39]. A standard marker (100–600 bp, produced by Shanghai Biocolor BioScience & Technolgy Company) was added on each gel as control during the gel run. The size of PCR products were detected by BIO Imagine System with software Genetools from SynGene and were manually re-checked twice [31]. The length of each allele was compared to the standard bands of the standard marker and scored.

### Data analysis

Means and standard deviation (SD) for 12 traits were calculated using Excel software. The percentage of phenotypic variation explained by population structure was calculated using a General Linear Model (GLM) with software SPSS 17.0 for Windows (SPSS Inc. Chicago, IL, USA). The broad-sense heritability (*H^2^*) was calculated as *H^2^* = 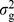/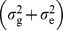, where 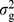 is the genetic variance, 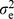 is the environmental variance. They were calculated using software QGA Station 1.0 (Zhu Jun, Zhejiang University, China). Correlation coefficients between traits were calculated using the software SPSS.

Polymorphism information content (PIC) which measures the extent of polymorphism for marker gene(s) or marker sequence(s) was calculated using the program POWERMARKER V3.25. Software Structure V2.3.1 was used to infer population structure and get Q matrices [40], [41]. During the running, a range of genetic clusters from *K* = 1 to 15 with the admixture model was examined, and for each K it was replicated 5 times. Each run implemented with a burn-in period of 100,000 steps followed by 100,000 Monte Carlo Markov Chain replicates. Due to the distribution of L(*K*) did not show a clear cutoff point for the true *K*, an ad hoc measure Δ*K* was used to detect the numbers of subgroup. That run with the maximum likelihood was applied to subdivide the varieties into different subgroups based on the maximum membership probability. A Q-matrix was obtained from the membership probability of each variety. Our previous study indicated that there were two distinct subgroups in Panel 1, which were in accordance with the germplasm types of *indica* and *japonica* rice [31]. The Q-matrix was used for further association mapping. The Loiselle algorithm was chosen for calculating kinship matrix (K) by software SPAGeDi [42]. Rare alleles with frequency of less than 10% in population were filtered as missing data in association analysis. Quantile–quantile plots were generated for observed against expected −log_10_ (*P*) using software SAS version 9.0 (SAS Institute 2002), where observed *P* values were obtained from association mapping and expected *P* values from the assumption that no associations happened between marker and trait.

Association analysis was performed using the software TASSEL (www.maizegenetics.net/tassel). For the mixed linear model (MLM) method, both K and Q matrices were incorporated, whereas for the GLM method, only population structure information (Q-matrix) was used as a covariate. Significance of associations between loci and traits were determined by their *P* values (*P*<0.05) which were calculated by the statistical models, and the phenotypic variance explained by the significant loci was calculated through analysis of variance (ANOVA). Since MLM method performs better in controlling spurious associations than GLM method [43], we first ranked the significant (*P*<0.05) association from MLM and then compared the significance of these markers (*P*<0.05) in the permutation based on GLM association tests. For the comparison, we calculated and used other two significant thresholds (i.e. Minimum Bayes factor (BF) and Bonferroni threshold) besides the *P* value. BF was calculated using the following formula: BF = −e*P*ln(*P*) [44], [45]. The Bonferroni threshold [46] was 1/274 = 0.00365, where 274 is the number of association tests for each traits in this study. Duncan multiple comparisons was implemented in SPSS for comparisons of performance of agronomic traits relevant to different alleles of the significant trait-marker associations.

## Results

### Phenotypic variation

The rice landraces in Panel 1 revealed a wide range of phenotypic variation in 12 agronomic traits (Table 1). Heading date, plant height, 1000-grain weight, flag leaf length, flag leaf length/width, and panicle numbers per plant showed similar distributions in both two years (Figures S1–S6 in File S1). On average about 12.4% of phenotypic variation was influenced by population structure. The broad-sense heritability ranged from 74.8% (1000GW) to 99.8% (GW) for these traits.

**Table 1:** Descriptive statistics, percentage of phenotypic variation explained by population structure (*R^2^*), and heritability in broad sense (*h^2^*) for 12 agronomic traits in Panel 1.

Trait	Year	Mean±S.D.	Range	*R^2^*(%)	*h^2^*(%)
Heading days (day)	2008	71.0±7.6	61.0–95.0	1.2	78.1
	2009	66.7±9.9	52.0–92.0	7.5	
Plant height (cm)	2008	144.5±26.4	66.0–209.5	25.1	97.4
	2009	150.8±30.3	72.8–229.0	28.1	
Seed set rate (%)	2008	79.1±11.7	24.4–98.0	1.2	76.8
	2009	84.3±11.5	25.3–98.3	10.8	
Panicle length (cm)	2008	24.8±2.9	15.8–31.5	24.9	94.9
	2009	25.6±3.3	15.7–35.2	31.5	
Grain length(GL) (mm)	2008	7.9±0.6	6.2–9.6	9.6	76.5
	2009	8.0±0.6	6.6–10.5	5.3	
Grain width(GW) (mm)	2008	3.1±0.4	2.3–4.1	8.2	99.4
	2009	3.1±0.3	2.4–3.7	12.0	
GL/GW	2008	2.6±0.4	1.9–3.9	10.5	99.5
	2009	2.6±0.4	1.9–3.7	11.1	
1000-grain weight (g)	2008	21.5±3.8	11.0–34.1	1.8	74.8
	2009	23.0±3.9	11.8–35.7	2.9	
Flag leaf length(FLL) (cm)	2008	43.2±8.6	23.0–75.0	34.1	88.7
	2009	39.6±6.6	23.6–56.1	17.5	
Flag leaf width(FLW) (cm)	2008	1.7±0.3	1.0–2.2	2.6	99.8
	2009	1.6±0.3	0.9–2.2	6.7	
FLL/FLW	2008	26.3±6.4	13.5–50.2	28.4	97.2
	2009	25.1±6.2	13.6–49.0	2.5	
Panicles number per plant	2008	7.9±2.6	3.0–20.0	12.3	94.6
	2009	8.7±2.5	4.6–18.2	0.6	

### Phenotypic correlation analysis

Extremely significant (*P*<0.01) positive correlations both in 2008 and 2009 were found between HD and PH, PH and PL, FLL and FLL/FLW, PL and FLL, PL and FLW, GL and GL/GW, GW and 1000GW, GL and 1000GW, HD and FLW, PH and FLL, SS and 1000GW, PH and FLW (Table 2). Extremely significant (*P*<0.01) negative correlations in both two years were found between HD and 1000GW, GW and GL/GW, FLW and FLL/FLW, FLW and PN.

**Table 2:** Correlation coefficients for 12 agronomic traits in 2008 and 2009.

	HD	PH	SS	PL	GL	GW	GL/GW	1000GW	FLL	FLW	FLL/FLW	PN
HD		0.263**	−0.085	0.072	−0.017	0.028	−0.040	−0.316**	0.168	0.497**	0.034	−0.103
PH	0.425**		0.107	0.544**	0.104	0.112	−0.092	0.189*	0.477**	0.240**	0.224**	−0.124
SS	−0.176*	0.005		0.074	−0.140	−0.005	−0.083	0.301**	0.074	0.146	−0.044	−0.151
PL	0.364**	0.648**	0.013		0.146	0.014	0.024	0.143	0.378**	0.294**	0.116	−0.074
GL	0.031	0.018	−0.022	0.254**		0.014	0.515**	0.247**	0.153	−0.014	0.116	−0.080
GW	−0.076	0.120	0.007	−0.035	−0.363**		−0.836**	0.233**	−0.010	0.000	0.015	−0.040
GL/GW	0.064	−0.112	−0.016	0.135	0.758**	−0.864**		−0.103	0.043	−0.010	0.015	−0.006
1000GW	−0.325**	−0.003	0.255**	0.098	0.280**	0.514**	−0.263**		0.307**	0.089	0.168	−0.157
FLL	0.064	0.243**	0.014	0.337**	0.070	0.072	−0.012	0.149		0.098	0.732**	−0.202*
FLW	0.216**	0.291**	0.016	0.282**	−0.014	−0.042	0.035	−0.073	0.097		−0.569**	−0.324**
FLL/FLW	−0.129	−0.034	0.021	−0.037	−0.022	0.133	−0.106	0.129	0.591**	−0.681**		0.042
PN	−0.079	−0.120	0.116	−0.133	0.167*	−0.107	0.160	0.021	−0.051	−0.404**	0.286**	

Note: Above the diagonal is the Pearson correlation coefficient in 2008 and below the diagonal there is the Pearson correlation coefficient in 2009. HD: Heading days, PH: Plant height, SS: Seed set rate, PL: Panicle length, GL: Grain length, GW: Grain width, 1000GW: 1000-grain weight, FLL: Flag leaf length, FLW: Flag leaf width and PN: Panicles number per plant.

**,* represents significant correlation at α = 0.01 and 0.05, respectively.

### Relative kinship among individuals in the three panels

**Figure 1:**
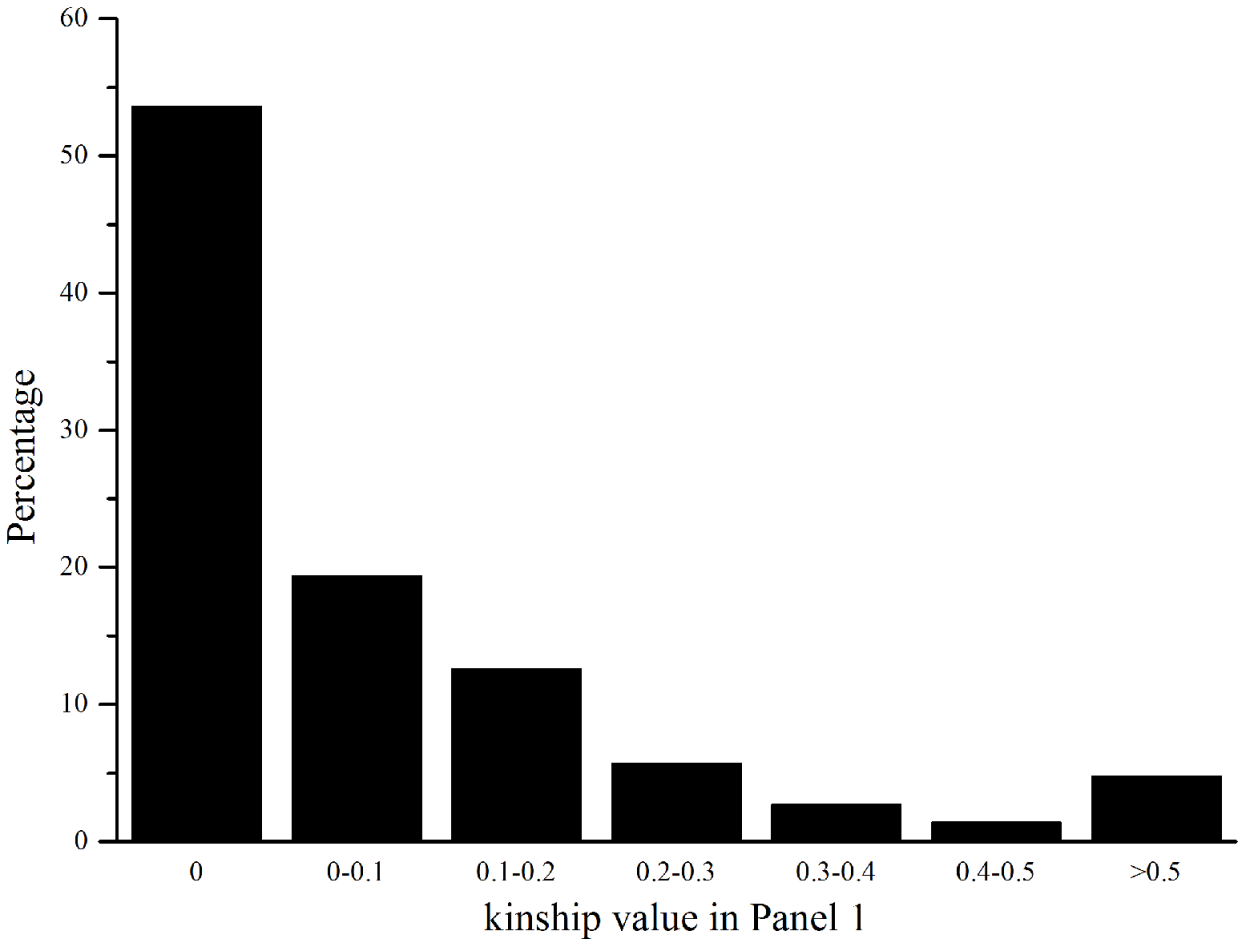
Distribution of pairwise relative kinship values in Panel 1. The height of the black bar represents the percentage of varieties in different ranges of kinships.

In Panel 1, about 55% of pairwise kinship estimates were zero and only 4.73% of pairwise kinship coefficient were larger than 0.5, indicating that these varieties were unrelated (Figure 1). In Panel 2 and 3, 55.9% and 60.4% of pairwise kinship coefficient were larger than 0.5, respectively (Figure S7 in File S1), indicating that these varieties have certain kinship relationship.

### The effect of controlling type I error using MLM

**Figure 2:**
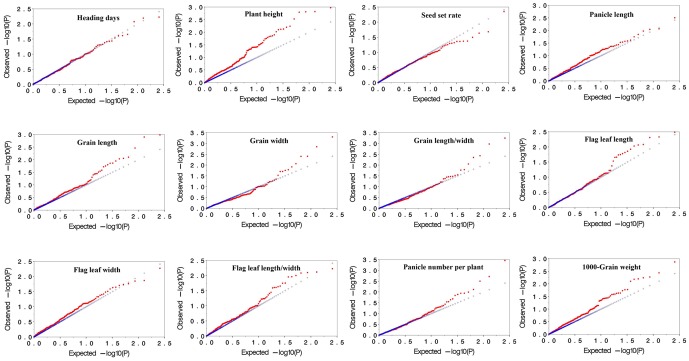
Plots of observed versus expected *P*-values using MLM (Q+K) model for 12 agronomic traits in 2008. The blue symbol the represents expected *P*-values, and the red symbol represents the observed *P*-values.

**Figure 3:**
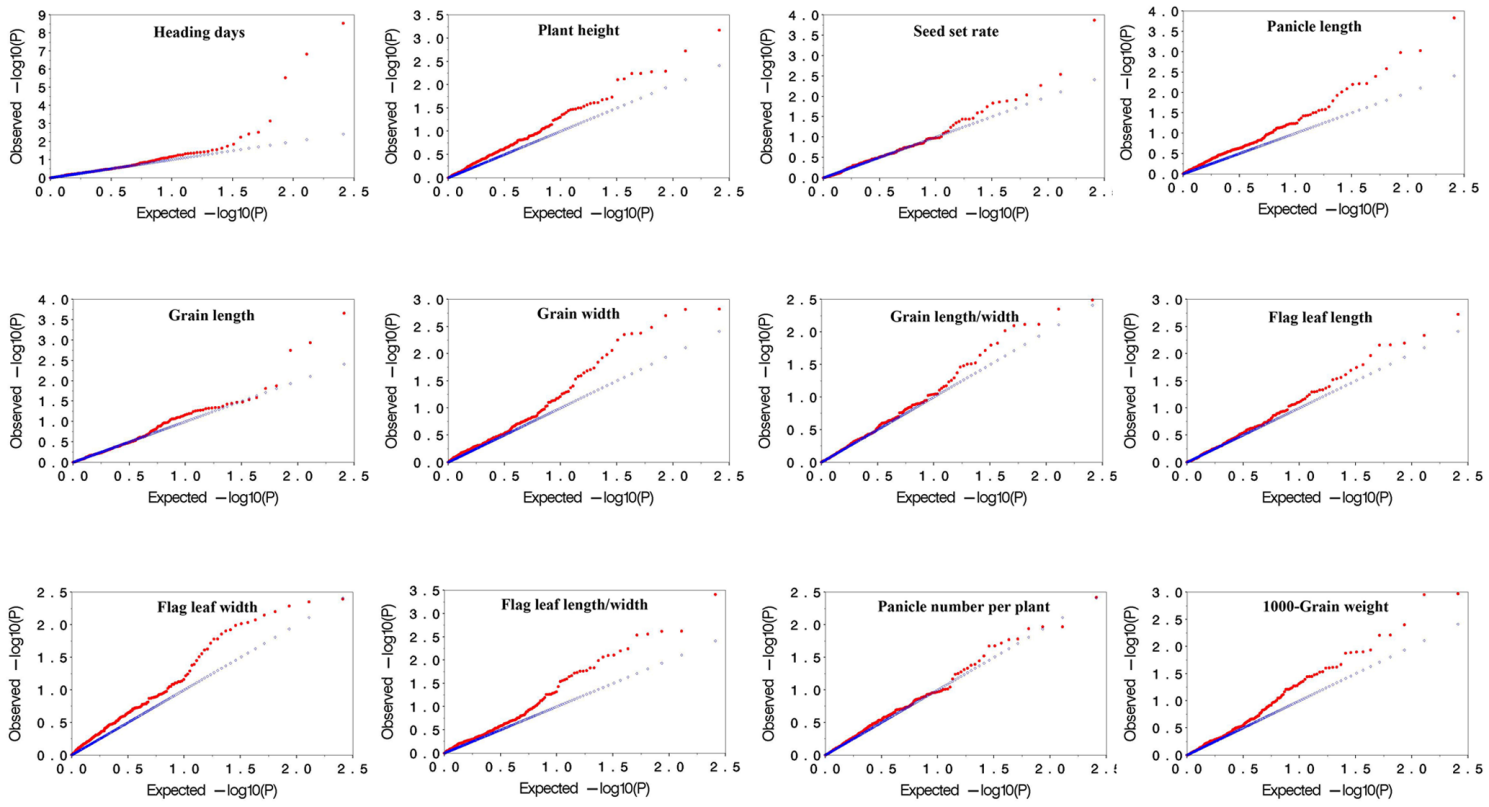
Plots of observed versus expected *P*-values using MLM (Q+K) model for 12 agronomic traits in 2009. Blue symbol represents expected *P*-values, and red symbol represents observed *P*-values.

Observed versus expected *P* values for each trait-marker association were plotted to assess the control of type I errors. Uniform distributions between the observed and expected *P* values for all traits were observed, and were demonstrated by similar distributions in two years (Figures 2 and 3). As the deviations from the expectation demonstrated that the statistical analysis may cause spurious associations [28], our result indicated that the false positives were well controlled in the MLM method in this study.

### Trait-marker associations

152 significant (*P*<0.05) trait-marker associations were found using the GLM model for the 12 agronomic traits both in 2008 and 2009, and 15 (∼10%) of 152 trait-marker associations were detected in the previous studies (Table 3). Furthermore, 184 and 217 significant (*P*<0.05) trait-marker associations were identified using MLM in 2008 and 2009, respectively. Among them, 76 trait-marker associations were significant (*P*<0.05) both in 2008 and 2009. The number of significant loci associated with each agronomic trait in two years ranged from 0 (seed set rate) to 13 (plant height). Moreover, 24 (∼32%) of the 76 trait-marker associations were in the same or similar genomic regions where QTLs were detected in previous studies (http://www.gramene.org/), and the other 52 trait-marker associations were new associations which were not previously identified.

**Table 3:** Summary of association mapping results for 12 agronomic traits using MLM model in Panel 1.

Traits	No.^a^	No. of significant associations using MLM		
		2008	2009	No.^c^
HD	4(2)^b^	11(6)	15(8)	10(0)
PH	13(10)	20(14)	16(12)	21(5)
SS	0(0)	9(2)	15(4)	1(0)
PL	2(2)	18(11)	24(13)	11(2)
GL	10(3)	19(4)	13(2)	9(1)
GW	6(0)	13(1)	18(4)	2(0)
GL/GW	9(2)	14(3)	14(3)	9(2)
1000GW	8(3)	22(12)	24(12)	15(1)
FLL	5(0)	14(2)	16(4)	4(0)
FLW	10(1)	13(2)	22(4)	38(2)
FLL/FLW	8(0)	15(1)	25(0)	23(1)
PN	1(1)	16(7)	15(9)	9(1)
Total	76(24)	184(65)	217(75)	152(15)

Note: In this table,

a number of SSR loci shows the same trait-marker association (MLM, *P*<0.05) in the both years;

b number in parentheses represents the number of trait-marker associations which is located in the same or similar genomic region where QTLs were detected in previous studies;

c the number of SSR loci showing the same trait-marker association (GLM, *P*<0.05) in both years.

HD: Heading days, PH: Plant height, SS: Seed set rate, PL: Panicle length, GL: Grain length, GW: Grain width, 1000GW: 1000-grain weight, FLL: Flag leaf length, FLW: Flag leaf width and PN: Panicles number per plant.

Eleven of the 76 trait-marker associations had 10% or more explained percentage of the total variation (*R^2^*), i.e. HD (PSM184), PH (RM530, RM590), PL (PSM184), GL/GW (RM447), FLL (RM287), FLW (RM235), 1000GW (RM7, RM538 and RM206), and PN (RM311) both in 2008 and 2009 (Table 4). When using BF and the Bonferroni threshold as significance thresholds, there were 15 and 3 trait-marker associations out of the 76 significant associations which still showed significant associations, respectively. Moreover, the three trait-marker significant associations shown by Bonferroni threshold were also significant when using BF as significant threshold. Furthermore, 59 of the 76 trait-marker associations were found to be significant when using the GLM model in two years.

**Table 4:** Association mapping results for 12 agronomic traits in two years using MLM model in Panel 1.

No.	Marker names	Chr. No	Chr. position(cM)	2008		2009		QTL Accession ID^f^	QTL region (cM)
				*P* value (MLM)	*R^2^* (%)	*P* value (MLM)	*R^2^* (%)		
Heading days									
1	RM341	2	82.7	0.0382^e^	3.29	0.0037^a^ ^c^ ^e^	6.18	AQFW189	81.0–129.6
2	RM339	8	72.2	0.0469^c^ ^e^	4.72	0.0291^e^	5.16	AQS010	45.4–73.0
3	RM224	11	120.1	0.0246^c^	8.72	0.0176^c^	8.81	-	-
4	PSM184	12	26.0	0.0289	11.08	0.0001^b^ ^c^ ^d^	35.4	-	-
Plant height									
1	RM530	2	170.1	0.0015^b^ ^c^ ^d^ ^e^	12.06	0.0007^b^ ^c^ ^d^ ^e^	12.16	AQCU198	165.4–189.4
2	RM138	2	196.8	0.0074^a^ ^c^ ^e^	5.74	0.0018^b^ ^c^ ^d^ ^e^	6.93	CQV7	189.9–202.8
3	PSM130	3	130.7	0.0395^c^ ^e^	4.88	0.0340^c^ ^e^	4.62	CQAB22	94.3–132.8
4	RM469	6	2.2	0.0094^a^ ^c^ ^e^	10.86	0.0241^e^	7.98	AQFW082	0–17.3
5	RM204	6	25.1	0.0146^c^ ^e^	9.51	0.0050^a^ ^c^ ^e^	10.37	AQHR045	24.1–37.9
6	RM225	6	26.2	0.0449^e^	6.10	0.0497^e^	5.35	AQHR045	24.1–37.9
7	RM18	7	90.4	0.0010^b^ ^c^ ^d^	8.26	0.0314^c^	3.16	-	-
8	RM219	9	11.7	0.0029^b^ ^c^ ^d^ ^e^	10.82	0.0360^c^ ^e^	5.86	AQGS003	0–23.8
9	PSM167	10	55.6	0.0478^c^	2.93	0.0074^a^ ^c^	4.92	-	-
10	RM147	10	99.8	0.0462^e^	7.20	0.0392^e^	8.27	AQFF070	90.4–106.0
11	RM590	10	117.2	0.0078^a^	14.89	0.0056^a^	14.08	-	-
12	RM21	11	85.7	0.0396^e^	7.66	0.0439^e^	6.82	AQHR060	80.8–87.7
13	PSM184	12	26.0	0.0015^b^ ^c^ ^d^ ^e^	16.95	0.0424^e^	9.01	AQFW086	14.9–44.1
Panicle length									
1	RM228	10	96.3	0.0404^c^ ^e^	3.40	0.0313^c^ ^e^	3.20	AQCU037	88.0–129.0
2	PSM184	12	26.0	0.0424^c^ ^e^	10.80	0.0009^b^ ^c^ ^d^ ^e^	16.44	AQFJ050	0–28.1
Grain length									
1	RM81A	1	77.5	0.0307^c^	5.26	0.0357^c^	4.75	-	-
2	RM341	2	82.7	0.0078^a^ ^c^	5.36	0.0017^b^ ^c^ ^d^	7.08	-	-
3	RM156	3	125.7	0.0010^b^ ^c^ ^d^	8.30	0.0002^b^ ^c^ ^d^	10.10	-	-
4	RM127	4	150.1	0.0352^c^ ^e^	5.05	0.0480^e^	4.33	CQAL24	149.7–173.2
5	PSM158	9	33.0	0.0112^c^ ^e^	6.84	0.0372^c^ ^e^	4.69	AQCA004	0–63.4
6	PSM171	11	4.8	0.0284^c^ ^e^	5.38	0.0420^c^ ^e^	4.52	AQT016	0–72.7
7	RM144	11	123.2	0.0012^b^ ^c^ ^d^	8.01	0.0288^c^	3.40	-	-
8	RM4A	12	5.2	0.0399^c^	6.33	0.0450^c^	5.77	-	-
9	RM277	12	57.2	0.0190^c^	9.01	0.0011^b^ ^c^ ^d^	13.42	-	-
10	PSM191	12	99.7	0.0359^c^	5.05	0.0470^c^	4.37	-	-
Grain width									
1	RM237	1	115.2	0.0162^c^	4.40	0.0019^b^ ^c^ ^d^	6.95	-	-
2	RM276	6	40.3	0.0013^b^ ^c^ ^d^	8.14	0.0015^b^ ^c^ ^d^	7.50	-	-
3	RM557	6	41.9	0.0038^a^ ^c^	8.67	0.0032^a^ ^c^ ^d^	8.53	-	-
4	RM223	8	80.5	0.0005^b^ ^c^ ^d^	9.50	0.0086^a^ ^c^	4.96	-	-
5	RM447	8	124.6	0.0195^c^	9.12	0.0014^b^ ^c^ ^d^	13.13	-	-
6	RM19	12	20.9	0.0085^a^ ^c^	9.05	0.0044^a^ ^c^	9.58	-	-
GL/GW									
1	RM237	1	115.2	0.0036^a^ ^c^ ^d^	6.38	0.0158^c^	4.06	-	-
2	RM208	2	186.4	0.0299^c^	8.07	0.0191	9.54	-	-
3	RM559	4	155.8	0.0169^c^	7.69	0.0343^c^	6.04	-	-
4	RM276	6	40.3	0.0161^c^ ^e^	4.39	0.0421^c^ ^e^	2.91	CQAL18	18.5–49.0
5	RM557	6	41.9	0.0043^a^ ^c^ ^e^	8.26	0.0307^c^ ^e^	4.89	CQAL18	18.5–49.0
6	RM223	8	80.5	0.0006^b^ ^c^ ^d^	9.11	0.0075^a^ ^c^	4.99	-	-
7	RM447	8	124.6	0.0087^a^	10.40	0.0032^a^ ^c^ ^d^	11.41	-	-
8	RM224	11	120.1	0.0357	7.73	0.0044^a^ ^c^	10.80	-	-
9	RM277	12	57.2	0.0329^c^	7.89	0.0296^c^	7.54	-	-
Flag leaf length									
1	RM138	2	196.8	0.0092^a^ ^c^	5.34	0.0401	3.14	-	-
2	RM469	6	2.2	0.0182^c^	9.40	0.0287^c^	8.14	-	-
3	PSM340	9	90.1	0.0046^a^ ^c^	6.02	0.0272^c^	3.55	-	-
4	RM167	11	20.3	0.0085^a^ ^c^	7.17	0.0063^a^ ^c^	7.49	-	-
5	RM287	11	68.6	0.0032^a^ ^c^ ^d^	12.22	0.0045^a^ ^c^	11.28	-	-
Flag leaf width									
1	PSM128	3	96.6	0.0326^c^	3.52	0.0242^c^	3.70	-	-
2	RM571	3	205.4	0.0417^c^ ^e^	4.92	0.0091^a^ ^c^ ^e^	6.91	AQFW011	190.5–209.0
3	RM225	6	26.2	0.0172^c^	7.95	0.0353^c^	6.28	-	-
4	RM584	6	26.2	0.0397^c^	3.26	0.0123^c^	4.57	-	-
5	RM182	7	61.0	0.0224	5.90	0.0277^c^	5.25	-	-
6	RM407	8	5.7	0.0257^c^	8.67	0.0095^a^ ^c^	11.53	-	-
7	RM339	8	72.2	0.0197^c^	6.11	0.0040^a^ ^c^	8.18	-	-
8	RM222	10	11.3	0.0053^a^ ^c^	6.07	0.0062^a^ ^c^	5.49	-	-
9	PSM170	10	68.6	0.0181^c^	8.68	0.0138^c^	8.62	-	-
10	RM235	12	91.3	0.0140^c^	16.67	0.0308^c^	13.71	-	-
FLL/FLW									
1	RM571	3	205.4	0.0246^c^	5.78	0.0028^a^ ^c^ ^d^	8.58	-	-
2	RM348	4	137.9	0.0231^c^	7.45	0.0056^a^ ^c^	9.20	-	-
3	RM559	4	155.8	0.0499	6.07	0.0004^b^ ^c^ ^d^	13.68	-	-
4	RM153	5	3.0	0.0388^c^	5.05	0.0172^c^	5.88	-	-
5	RM469	6	2.2	0.0108^c^	10.66	0.0225^c^	8.34	-	-
6	PSM340	9	90.1	0.0078^a^ ^c^	5.51	0.0146^c^	4.29	-	-
7	PSM172	11	7.6	0.0110^c^	8.77	0.0027^b^ ^c^ ^d^	10.40	-	-
8	RM167	11	20.3	0.0058^a^ ^c^	8.10	0.0023^b^ ^c^ ^d^	8.86	-	-
1000GW									
1	RM341	2	82.7	0.0036^a^ ^c^ ^d^	6.89	0.0128^c^	4.49	-	-
2	PSM374	2	83.6	0.0078^a^ ^c^	7.92	0.0400^c^	4.69	-	-
3	RM7	3	64	0.0052^a^ ^c^ ^e^	13.92	0.0125^c^ ^e^	10.77	AQDV056	51.3–86.7
4	RM252	4	99	0.0256^c^	7.53	0.0011^b^ ^c^ ^d^	12.00	-	-
5	RM538	5	132.7	0.0366	8.51	0.0321^c^	12.38	-	-
6	RM447	8	124.6	0.0331^c^	9.03	0.0355^c^	7.83	-	-
7	RM239	10	25.2	0.0253^c^ ^e^	8.51	0.0295^c^ ^e^	7.85	AQEY016	21.1–44.4
8	RM206	11	102.9	0.0013^b^ ^c^ ^d^ ^e^	9.03	0.0115^c^ ^e^	12.19	AQGP076	102.9–102.9
Panicle number per plant									
1	RM311	10	25.2	0.0031^b^ ^c^ ^d^ ^e^	17.52	0.0190^c^ ^e^	12.42	AQDY128	20.9–25.9

Note:

a BFmin with moderate to strong evidence for association (>0.05–0.13);

b BFmin with strong to very strong evidence for association (≤0.05);

c supported by the GLM in TASSEL (≤0.05);

d the Bonferroni threshold (<0.0036);

e supported by previous literature; *R^2^* represents the genetic variants explained by the marker; QTLs detected in previous studies (http://www.gramene.org/).

### Impact of allele frequency on the power to detect a QTL

**Figure 4:**
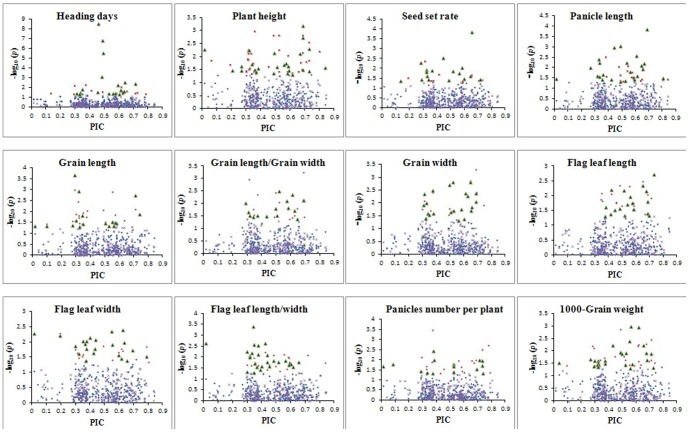
Relationship between PIC and *P*-value for marker–trait associations for 12 agronomic traits in two years. Green asterisk refers to the total markers used in traits in 2008. A red asterisk refers to the markers significantly associated with traits in 2008. A purple asterisk refers to the total markers used in traits in 2009. A green triangle refers to the markers significantly associated with traits in 2009.

We further investigated the relationship between the *P* values of significant trait-marker associations and the PIC values of related markers. For all trait-marker associations, only 3.5% of markers had a PIC value lower than 0.2 (Figure 4). Most of the markers which showed significant associations with related traits had a PIC value larger than 0.2, which meant that these markers showed a higher power to detect a QTL.

### Verification of association mapping results in Panel 2 and Panel 3

For the 76 significant trait-marker associations in Panel 1, because some SSR markers show more than one significant associations with related traits, the number of related SSR markers is less than 76, i.e. 55 SSR markers in this study. All these 55 SSR markers were further used to genotype Panel 2 and 3. Based on these genotyping data, the population structure of both Panel 2 and 3 indicated two distinct subgroups (Figure S8 in File S1).

Association analysis was performed within the two Panels using both MLM and GLM approaches with the 55 SSR markers. A total of 20 and 31 significant trait-marker associations were detected using MLM within Panel 2 and Panel 3, respectively. Seven significant trait-marker associations which were detected in Panel 1 using MLM model were identical with those in Panel 2 and Panel 3 using the GLM model, respectively. However, there was no identical trait-marker association within the three Panels when using the MLM model (Table 5). In Panel 2, RM219 [47], RM469 [48] and RM204 [49] showed significant associations with plant height and they were also reported by previous researches. Among them, the association for marker RM469 with plant height had the highest *R^2^* (10.08%). Similarly, in Panel 3, the association for marker RM590 with plant height had the highest *R^2^* (39.96%). RM339 which showed significant associations with heading days, were reported by previous researches [50] (Table 6).

**Table 5:** Summary of trait-marker associations within the three Panels.

Population	MLM(1)	GLM(2)	GLM(3)
Panel 2	0	7(3)	3(1)
Panel 3	0	7(2)	2(1)

Note:

(1) Number of the same trait-marker associations using MLM found both in Panel 1 and Panel 2 or Panel 3;

(2) Number of the same trait-marker associations using GLM (*P*<0.05) found both in Panel 1 and Panel 2 or Panel 3.

(3) Number of the same trait-marker associations using GLM (*P*<0.01) found both in Panel 1 and Panel 2 or Panel 3.

In parentheses, the number of trait-marker associations which are identical with the published mapping results in previous literature is given.

**Table 6:** The same trait-marker associations in Panel 2 and 3 using GLM model compared with those in Panel 1.

Trait	Panel 2						Panel 3					
	Marker names	Chr. No	Genetic distance (cM)	*R^2^* (%)	QTL Accession ID	QTL region (cM)	Marker names	Chr. No	Genetic distance (cM)	*R^2^* (%)	QTL Accession ID	QTL region (cM)
Plant height	RM204a	6	25.1	4.79	AQHR045	24.1–37.9	RM590	10	117.2	39.96	AQEX006	113.5–117.0
	RM219a	9	11.7	6.46	AQGS003	0–23.8						
	RM469a	6	2.2	10.08	AQFW082	0–17.3						
Grain length	PSM158a	9	33.0	4.69	AQCA004	0–63.4	PSM191	12	99.7	11.42	AQCV009	47.0–51.5
GL/GW	RM559	4	155.8	3.73	AQFA016	47.8–47.8	RM208	2	186.4	6.74	AQGB055	55.9–77.9
							RM277	12	57.2	5.44	AQCV021	30.0–39.7
Flag leaf length	RM138	2	196.8	7.34	AQFW023	205.8–231.5						
Heading days							RM339a	8	72.2	9.00	AQS010	45.4–73.0
							PSM184	12	26.0	3.92	AQAX020	17.5–22.2
FLL/FLW	RM469	6	2.2	9.90	AQEJ014	33.7–38.3						
1000-GW							PSM374	2	83.6	4.47	AQT007	0–81.4

Note: ^a^represents the locus identical with previous mapping results, *R^2^* represents the genetic variance explained by the marker.

### Performance of traits relevant to different alleles of significant loci

Seven markers, i.e. PSM184, RM447, RM469, RM235, RM206, RM311, and RM277, were selected for analysis of trait performance relevant to different alleles of significant loci based on their high explained percentage of genetic variation and supported by several significant thresholds (Table 4). For PSM184, the individuals carrying the allele 222 bp (the size of PCR product for the SSR markers, the same as below) had a significantly (*P*<0.01) lower plant height and panicle length than those carrying other two alleles 205 bp and 215 bp (Table 7). For RM447, the individuals carrying the allele 109 bp had a significantly (*P*<0.01) higher grain width and significantly (*P*<0.01) lower grain length/width ratio than those carrying other two alleles 100 bp and 117 bp. For RM469, the individuals carrying the allele 94 bp had a significantly (*P*<0.01) lower flag leaf length than those carrying other two other alleles 83 bp and 88 bp. For RM206, the individuals carrying the allele 162 bp had a significantly (*P*<0.01) higher 1000-grain weight than those carrying the other four alleles 123 bp, 125 bp, 130 bp and 143 bp. For RM311, the individuals carrying the allele 143 bp, 143 bp and 153 bp showed a significantly (*P*<0.05) higher panicle number per plant than those carrying other two alleles 147 bp and 157 bp. For RM235, the individuals carrying the allele 108 bp showed a significantly (*P*<0.05) higher flag leaf width than those carrying the alleles 115 bp, 117 bp, 121 bp and 123 bp, whereas the individuals carrying the allele 123 bp had a significantly (*P*<0.05) lower flag leaf width than those carrying the alleles 91 bp, 108 bp, and 115 bp. For RM277, the individuals carrying the allele 117 bp had a higher grain length than those carrying the allele 111 bp (Duncan multiple comparisons was not been performed due to it had only two alleles).

**Table 7:** Duncan multiple comparisons for different allelic effects on traits.

Trait	Marker	Allele (bp)	Mean±SD	Trait	Marker	Allele (bp)	Mean±SD
Plant height (cm)	PSM184	205	148.96±26.94^Aa^	1000-grain weight (g)	RM206	123	21.76±4.48^Aa^
		215	152.34±28.01^Aa^			125	21.80±2.38^Aa^
		222	134.11±32.91^Bb^			130	22.39±3.94^Aa^
Panicle length (cm)	PSM184	205	25.52±3.14^Aa^			143	20.71±3.51^Aa^
		215	25.37±3.13^ABa^			162	26.09±5.30^Bb^
		222	23.89±4.12^Bb^	Panicle number per plant	RM311	143	8.14±2.77^Aa^
Grain width (mm)	RM447	100	3.06±0.31^Aa^			145	8.66±2.32^Aa^
		109	3.24±0.40^Bb^			147	7.86±2.18^Ab^
		117	3.05±0.36^Aa^			153	8.75±3.18^Aa^
Grain length/width	RM447	100	2.66±0.32^Aa^			157	7.54±2.10^Ab^
		109	2.44±0.44^Bb^	Flag leaf width (cm)	RM235	91	1.79±0.19^Aab^
		117	2.69±0.45^Aa^			108	1.82±0.21^Aa^
Flag leaf length (cm)	RM469	83	42.02±7.47^Aa^			115	1.70±0.18^Ab^
		88	43.08±8.13^Aa^			117	1.63±0.35^Abc^
		94	39.10±7.84^Bb^			121	1.59±0.28^Abc^
						123	1.57±0.25^Ac^

Note: Capital and lower letters represent significant difference at α = 0.01 and 0.05, respectively. Allele (bp) is PCR product amplified by SSR markers.

## Discussion

### Comparison of different mapping populations for association mapping

An appropriate population with maximized phenotypic variation is critical for the success of an association analysis [18], [51]. Rice landraces represent an intermediate stage in domestication between wild and elite cultivars [2], which possess high genetic diversity and many exotic genes, and therewith provide useful germplasm resources for rice breeding. Moreover, association mapping based on a core collection of rice landraces would help to catch as much phenotypic variation as possible.

China is well known as one of the origin center of cultivated rice with abundant genetic resources for rice. As early as in 1920–1964s, Professor Ying Ting collected more than 7128 accessions of rice landraces from all over China as well as some countries which grow rice as a major crop. The collection is one of the earliest collections for rice germplasm resources and therefore was named Ting's rice germplasm collection [30]. Our previous results based on the core collection from it indicated that (1) the percentage of SSR loci pairs in significant (*P*<0.05) LD was 46.8%; (2) LD decayed rapidly to the threshold, i.e. the 95% quantile of *r* ^2^ between unlinked loci pairs, at 1.03 cM in the entire collection; and (3) there were many LD blocks. These previous results indicated that Panel 1 was an appropriate population for association mapping. Therefore, our association mapping was performed based on Panel 1.

The populations in previous studies for association analysis in rice included populations from the USDA core collection [14], [16], [24], landraces [16], [17], elite cultivars [16], and mini-core collection [23]. The mapping populations in the researches of Agrama et al. [14], [24], [52] and Li et al. [23] were subsets chosen randomly from the USDA core collection, which consisted of 92, 547 and 203 accessions, respectively. Moreover, the number of SSR markers was 123, 72 and 155, which was rather low for association mapping. In the study of Zhao et al. [11], 416 rice accessions including only two landraces were randomly selected and only 100 SSR markers were used.

Our results indicated that there is a wide-range of phenotypic variation for 12 agronomic traits in Panel 1. For heading days, flag leaf length, flag leaf width, grain length, grain width, grain length/width and panicle length, there was less phenotypic variations than described in the research of Jin et al. [16], while for plant height and 1000 grains weight, more phenotypic variation was found than reported in the research of Jin et al. [16]. The comparison with the results of Li et al. [23] indicated that less phenotypic variation was found in this study for heading days, 1000-grain weight and panicle length, while more was found for plant height, panicle number per plant and seed set rate. More phenotypic variation was found than reported in the research of Agrama et al. [14] for grain length, grain width and 1000-grain weight.

### Choice of statistical models and statistical parameters to control type I error

There are two frequently used models (i.e. MLM and GLM) which were implemented in the software TASSEL for association analysis [17], [23], [28]. In this study, we used the MLM (Q+K) [25] which accounted for population structure and kinship relationship to minimize spurious associations. For comparison, GLM was also used. In our study, 137 (∼90%) trait-marker associations were possibly new loci when using GLM model, whereas 52 (∼68%) trait-marker associations were possibly new loci when using MLM model. The ratio of possibly new significant loci detected using GLM model was much higher than that using MLM model. However, the new significant loci might be false positive because GLM model did not account for kinship.

Furthermore, the significance threshold (*P* value) must be set considerately in the association mapping. Using a smaller *P* value as threshold might lose more minor QTLs, while using a higher *P* value as threshold might get more false positive QTLs. To reliably interpret the MLM-derived significant associations in our study, we also used minimum BF estimation [44] for the MLM association results. Minimum BF estimates over *P* values of MLM approach may help to understand the overall impact of the associations [45]. We also used a Bonferroni threshold for identifying the associations derived from MLM analysis. The statistical parameters had been used successfully in association mapping of cotton [8]. Our results indicated that three significant trait-marker associations (i.e. plant height-RM530, grain length-RM156 and grain width-RM276) reached simultaneously the three thresholds (i.e. *P*<0.05, minimum BF, and the Bonferroni), which should be emphasized in future studies.

Moreover, molecular markers can be used to calculate the relative kinship between pairs of individuals in a study, which provides useful information for quantitative inheritance studies. Relative kinship reflects the approximate identity between two given individuals over an average probability of identity between two random individuals [25]. Our results indicated that most varieties had no or weak relationship with each other in the Ting's core collection, which might be due to the fact that these varieties were chosen from a diverse rice cultivating region including all over China, East Asia, and Southeast Asia. The quantile-quantile plot indicated that MLM (Q+K) performed well in association mapping on 12 agronomic traits, which could correct false positive trait-marker associations (Figure 2 and 3).

### Association analysis within Ting's core collection

Using Ting's rice core collection genotyped with 274 SSR markers, we performed association mapping for 12 agronomics traits with two years data using the MLM and GLM models implemented in TASSEL. In this study, most (∼80%) of the significant associations found using the MLM approach were also supported by the GLM approach in both years. The percentage of associations identical to previous reported QTLs was about 32%, which was higher than those in the research of Li et al. [23], but lower than those in the research of Agrama et al. [14]. The 76 significant trait-marker associations which were detected in both years were potential markers for effective marker-assisted selection programs in rice. Moreover, 52 of the 76 significant associations which were not detected in previous studies might be some new potential loci. For instance, the trait-marker associations for heading days with PSM184, plant height with RM590, grain length/width with RM447, flag leaf length with RM287, flag leaf width with RM235, 1000-grain weight with RM538, and 1000-grain weight with RM206, explained more than 10% of genetic variations both in 2008 and 2009.

For heading days, two of the four significant trait-marker associations were identical to previous reported QTLs, i.e. RM341 and RM339, were identical to previous reported QTLs in the research of Mei et al. [48] and Kunihiro et al. [50], respectively. Moreover, RM339 was also significantly associated with heading days in Panel 2 and 3. For heading days, ten of 13 significant trait-marker associations were identical to previous reported QTLs, i.e. RM530 in the research of Mei et al. [53], RM138 in the research of Fang et al. [51], PSM130 in the research of Cao et al. [54], RM469 (which also showed significant association in Panel 2 and 3) and PSM184 in the research of Mei et al. [48], RM204 (which also showed significant association in Panel 2 and 3) and RM225 in the research of Yang et al. [49], RM219 (which also showed significant association in Panel 2 and 3) in the research of Xiao et al. [47], RM21 and RM147 in the research of Lanceras et al. [55]. For panicle length, the two significant trait-marker associations were also identical to previous reported QTLs, i.e. RM228 and PSM184 in the research of Mei et al. [53] and Jiang et al. [56], respectively. For grain length, three of ten significant trait-marker associations were identical to previous reported QTLs in the previous researches, i.e. RM127 in the research of Tan et al. [57], PSM158 in the research of Xing et al. [58], and PSM171 in the research of Yoshida et al. [59]. For grain length/width, two of nine significant trait-marker associations were identical to previous reported QTLs in the previous researches, i.e. RM276 and RM557 reported by Tan et al. [57]. For flag leaf width, one of nine significant trait-marker associations were identical to previous reported QTLs, i.e. RM571 in the research of Mei et al. [48]. For 1000-grain weight, there of eight significant trait-marker associations were identical to previous reported QTLs in the previous researches, i.e. RM7 in the research of Hittalmani et al. [60], RM239 in the research of Gao et al. [61], and RM206 in the research of Cho et al. (this reference cannot be found, but QTL ID can be found in GRAMENE website). For panicle number per plant, the only one significant trait-marker association was also identical to previous reported QTL, i.e. RM311 in the research of Kobayashi et al. [62].

### Verification association mapping results within Panel 2 and Panel 3

It is worthwhile to further verify the significant associations identified within one population in a different population [29]. In this study, 55 SSR markers for the 76 trait-marker associations identified in Panel 1 were used to genotype two other populations, i.e. Panel 2 and Panel 3, and an association mapping was performed using both MLM and GLM approaches. When using the GLM approach, seven significant trait-marker associations were identical within Panel 1 and Panel 2 or Panel 3. Moreover, three of the seven identical significant trait-marker associations in the two panels were reported by previous studies. Although the GLM would bring more false positive results than the MLM when it was used alone, however, some significant trait-marker associations were first detected Panel 1 in our research and proved by several statistical thresholds as well as by previous mapping results. After that, we used the GLM to verify our mapping results in Panel 2 and 3. Therefore, it makes sense for verification of association mapping results by the fact that some common trait-marker associations were detected by the GLM approach.

We observed that there were no overlapping QTLs among the three panels with the GLM approach. The reasons might be (1) different compositions and origins of the varieties in three panels, where Panel 1 only consists of original rice landraces from China and some other rice growing countries which were collected during 1920–1964 before the emergence of hybrid rice, while Panel 2 consists of rice landraces as well as modern rice cultivars and maintainer lines in hybrid rice breeding from China, and Panel 3 is a worldwide collection and consists of modern rice cultivars including cytoplasmic sterile line, maintainer lines, and some landraces; (2) that different allelic frequencies might exist for the three panels which consist of different compositions and origins. The explanations were supported by our observations that (1) frequency of some alleles was different in the three panels and some alleles only exist in one panel (Table S3 in File S1), and (2) in our another experiment some alleles associated with aluminum tolerance were different for different germplasm types (data not shown).

When using the MLM approach, no identical significant trait-marker associations were found among the three panels. Previous studies on linkage mapping and association mapping also found that different mapping populations detected different QTL regions [14], [48], [63], [64], [65]. The reasons might be due to that (1) a much lower number of SSR markers (55 SSRs) was used in Panel 2 and Panel 3 than in Panel 1 (274 SSRs); (2) the 55 SSR markers are associated with relevant traits which were not randomly distributed across the genome, which might reduce the exactness of measurement for population structure and kinship; (3) the relative kinship calculated by 274 SSRs in Panel 1 was quite different than those calculated by the 55 SSRs in Panel 2 and 3, where in Panel 1 only 4.73% of pairwise kinship coefficient were larger than 0.5 and most of them were zero, whereas 55.9% and 60.4% of pairwise kinship coefficient in Panel 2 and 3 were larger than 0.5, respectively (Figure S8 in File S1); and (4) the degree of association might be reduced in MLM compared to those in GLM [50], which meant that when using much less SSR markers, the weak significant trait-marker associations in GLM might be not significant in MLM. As verification experiments were rarely performed in previous association studies, it is required to find an efficient solution for verification in future as well as to check the repeatability in different association mapping populations.

### Prospects for association mapping based on core collections

Association mapping has become a promising approach to mine elite genes within germplasm populations compared to traditional linkage mapping. Association mapping based on a core collection would help to capture as much phenotypic variation as possible. Compared to a natural population or a breeding population with a broad genetic basis, the LD level in a core collection might be low due to its diverse origin. Therefore, more markers might be required for association mapping. However, due to the quick LD decay, fine mapping using association analysis might be possible with a core collection. As quick, automated, economic genotyping technologies (such as genotyping by sequencing) have been developed, genotyping large germplasm resources with high density markers and GWAS in such mapping populations has become possible. Because such an association could be further applied in rice breeding by molecular marker assisted selection, it would be promising to make use of the elite genes in the diverse germplasm resources by the current strategy.

## Supporting Information

File S1 **Table S1,** Accessions, variety names, origin, germplasm types of 150 rice varieties in Panel 1. **Table S2,** Summary statistics of the 274 SSR markers used in this study. **Table S3,** Allele frequency of the 55 significant markers in three panels. **Figure S1,** Frequency distribution of heading days, plant height, seed set rate and panicle length in Panel 1 in 2008. The height of black bar represents the number of varieties in different range of traits. **Figure S2,** Frequency distribution of grain length, grain width, grain length/width and 1000 grain weight in Panel 1 in 2008. The height of black bar represents the number of varieties in different range of traits. **Figure S3,** Frequency distribution of flag leaf length, flag leaf width, flag leaf length/width and panicle number per plant in Panel 1 in 2008. The height of black bar represents the number of varieties in different range of traits. **Figure S4,** Frequency distribution of heading days, plant height, seed set rate and panicle length in Panel 1 in 2009. The height of black bar represents the number of varieties in different range of traits. **Figure S5,** Frequency distribution of grain length, grain width, grain length/width and 1000 grain weight in Panel 1 in 2009. The height of black bar represents the number of varieties in different range of traits. **Figure S6,** Frequency distribution of flag leaf length, flag leaf width, flag leaf length/width and panicle number per plant in Panel 1 in 2009. The height of black bar represents the number of varieties in different range of traits. **Figure S7,** Distribution of pairwise relative kinship values in Panel 2 and 3. The height of black bar represents the percentage of varieties in different range of kinships. **Figure S8,** Delta K change according to different K among Panel 2 and Panel 3 identified by STRUCTURE under Admixture model.
(DOC)
