# Supplementary material for: Association Mapping for Important Agronomic Traits in Core Collection of Rice (Oryza sativa L.) with SSR Markers
Source: PLoS One. 2014 Oct 31;9(10):e111508. doi: 10.1371/journal.pone.0111508 (PMC4216065; doi:10.1371/journal.pone.0111508)
Supplement: File S1 — Table S1, Accessions, variety names, origin, germplasm types of 150 rice varieties in Panel 1. Table S2, Summary statistics of the 274 SSR markers used in this study. Table S3, Allele frequency of the 55 significant markers in three panels. Figure S1, Frequency distribution of heading days, plant height, seed set rate and panicle length in Panel 1 in 2008. The height of black bar represents the number of varieties in different range of traits. Figure S2, Frequency distribution of grain length, grain width, grain length/width and 1000 grain weight in Panel 1 in 2008. The height of black bar represents the number of varieties in different range of traits. Figure S3, Frequency distribution of flag leaf length, flag leaf width, flag leaf length/width and panicle number per plant in Panel 1 in 2008. The height of black bar represents the number of varieties in different range of traits. Figure S4, Frequency distribution of heading days, plant height, seed set rate and panicle length in Panel 1 in 2009. The height of black bar represents the number of varieties in different range of traits. Figure S5, Frequency distribution of grain length, grain width, grain length/width and 1000 grain weight in Panel 1 in 2009. The height of black bar represents the number of varieties in different range of traits. Figure S6, Frequency distribution of flag leaf length, flag leaf width, flag leaf length/width and panicle number per plant in Panel 1 in 2009. The height of black bar represents the number of varieties in different range of traits. Figure S7, Distribution of pairwise relative kinship values in Panel 2 and 3. The height of black bar represents the percentage of varieties in different range of kinships. Figure S8, Delta K change according to different K among Panel 2 and Panel 3 identified by STRUCTURE under Admixture model. (DOC) [file pone.0111508.s001.doc]

**Table S1.** Accessions, variety names, origin, germplasm types of 150 rice varieties in Panel 1*

| Acc. | Variety name | Origin | *Indica* vs.  *Japonica* | Acc. | Variety name | Origin | *Indica* vs. *Japonica* |
| --- | --- | --- | --- | --- | --- | --- | --- |
| CC1 | Yin guang | Japan | *TJ* | CC76 | Guang ye hong mi | South China | *TI* |
| CC2 | Ao guo 5-B | Japan | *TJ* | CC77 | Da nuo | South China | *TI* |
| CC3 | Ai you | Japan | *TJ* | CC78 | Bai xu | South China | *TI* |
| CC4 | Tie geng yi shi ao | Yangtze River region | *TJ* | CC79 | Ya he | South China | *TI* |
| CC5 | Guo zhu | Japan | *TJ* | CC80 | Xu zai | South China | *TI* |
| CC6 | Ben dao | North China | *TJ* | CC81 | Dong an hou zi pu xiao he | Central China | *TI* |
| CC7 | Mang shui dao | Yangtze River region | *TJ* | CC82 | Tie gu pao | Central China | *TI* |
| CC8 | Bai mang gao li han dao bai | North China | *TJ* | CC83 | Chi mao zhan | South China | *TI* |
| CC9 | Jiu yue han | Northeast China | *TJ* | CC84 | Hu bei zao | Central China | *TI* |
| CC10 | Bi jie ma wei hong gu | Yunnan-Kweichow Plateau | *TJ* | CC85 | Ya jing mi | South China | *TI* |
| CC11 | Ai da tou | Yangtze River region | *TJ* | CC86 | Ba shi zi | Central China | *TI* |
| CC12 | Gui zao bai he | Yangtze River region | *TJ* | CC87 | Dong jun zi | Central China | *TI* |
| CC13 | Xiang dao | North China | *TJ* | CC88 | Early | Unknown | *TI* |
| CC14 | Zi jin gu | Northeast China | *TJ* | CC89 | Nuo | South China | *TI* |
| CC15 | Xiang chuan | Japan | *TJ* | CC90 | Gui zhao he 2 | Japan | *TI* |
| CC16 | Nagabo | Taiwan | *TJ* | CC91 | Hei nuo | Unknown | *TI* |
| CC17 | Bai ke da nuo | South China | *TJ* | CC92 | Da yi mao | Central China | *TI* |
| CC18 | San pai zhong | South China | *TJ* | CC93 | Gai cao zhan | Central China | *TI* |
| CC19 | Kai xuan | Japan | *TJ* | CC94 | Gamal | Unknown | *TI* |
| CC20 | Shi ban zhan | North China | *TJ* | CC95 | Bu gou wei | South China | *TI* |
| CC21 | He ke da nuo | South China | *TJ* | CC96 | Bai ke xi nuo | South China | *TI* |
| CC22 | Shen shui wan dao | Yangtze River region | *TJ* | CC97 | Ben dao | North China | *TI* |
| CC23 | Hong ben dao | Yangtze River region | *JC* | CC98 | Ba xian shu | Japan | *TI* |
| CC24 | Duan mang zi jin gu | Northeast China | *TJ* | CC99 | Guang hong mi dao | Yangtze River region | *TI* |
| CC25 | Bei jing jiang mi | North China | *JC* | CC100 | Wu mang yan guo qing | North China | *TI* |
| CC26 | Daeri | Celebes | *JC* | CC101 | Chang xu nuo | South China | *TI* |
| CC27 | Jian tou nuo | South China | *JC* | CC102 | Jiang wan 15 | Central China | *IC* |
| CC28 | Long you man dao | Yangtze River region | *TJ* | CC103 | Bai hua er | South China | *TI* |
| CC29 | Kun shan zhu zhou dao | Yangtze River region | *JC* | CC104 | Liu chang xian | South China | *TI* |
| CC30 | Huang ke zao 2 | Yangtze River region | *JC* | CC105 | Bai yin 3 | South China | *TI* |
| CC31 | Sheng fang da bai gu | North China | *JC* | CC106 | Shui zao huang pi | South China | *TI* |
| CC32 | Xiao dou | Japan | *JC* | CC107 | Yin 2 dong 7 | South China | *TI* |
| CC33 | Poetih | Celebes | *IC* | CC108 | Hou ma | South China | *TI* |
| CC34 | Tebaro | Sumbawa | *IC* | CC109 | Dong zhu 2 | South China | *TI* |
| CC35 | Ao hua da gui tou hong | Yangtze River region | *IC* | CC110 | Hong gen da mi | South China | *TI* |
| CC36 | Hui bei zi | Yunnan-Kweichow Plateau | *IC* | CC111 | Ben cheng guan yin zhan | Central China | *TI* |
| CC37 | Ba shi zi | Yangtze River region | *IC* | CC112 | Xi miao gu | South China | *IC* |
| CC38 | Zao sheng da ye | Japan | *IC* | CC113 | 186-zao guan yin zhan | Central China | *TI* |
| CC39 | Bnlastog | Low latitude region | *IC* | CC114 | Chang mang hei ma zao | Yunnan | *TI* |
| CC40 | Nuo mi | North China | *IC* | CC115 | Shui tian zhan gu nuo | South China | *TI* |
| CC41 | Xi chuan huang liu | South China | *TI* | CC116 | Chang han da hua ke | South China | *IC* |
| CC42 | Hei ju dao | Yangtze River region | *TI* | CC117 | Da he | South China | *TI* |
| CC43 | Guang fu 1 | Taiwan | *TI* | CC118 | Zeng cheng hei nuo | South China | *TI* |
| CC44 | Zhong qi jia qing | Yangtze River region | *IC* | CC119 | Ya he | South China | *TI* |
| CC45 | Ⅲ-49-4xi chuan huang | Taiwan | *IC* | CC120 | Bai gu zhan | Central China | *TI* |
| CC46 | Xin xian li | Yangtze River region | *TI* | CC121 | Die zhi | South China | *TI* |
| CC47 | Da liu tiao dao | Yangtze River region | *IC* | CC122 | You zhan | South China | *TI* |
| CC48 | Bai ke | South China | *TI* | CC123 | Chang sha wu qu wan dao | Central China | *TI* |
| CC49 | Chuan chi 1 | Central China | *TI* | CC124 | Tong ling hu nan xian | Central China | *TI* |
| CC50 | Tai nong 46 | Taiwan | *TI* | CC125 | Xiao mao dao | Central China | *TI* |
| CC51 | Ba chong sui | Japan | *TI* | CC126 | Jing xian si qu er gan | Central China | *TI* |
| CC52 | Yun nan bai | Central China | *TI* | CC127 | Zi xing er qu si dou xu | Central China | *TI* |
| CC53 | Liao yang ben di 4 | Northeast China | *IC* | CC128 | Chen hui fu dao | Central China | *TI* |
| CC54 | You zhan hong | South China | *TI* | CC129 | Bai gan zi | Central China | *TI* |
| CC55 | Hei nuo | South China | *TI* | CC130 | Han lu wei zhan | Central China | *TI* |
| CC56 | Xian zi zhan | Central China | *TI* | CC131 | Xin hua san qu tang mao zhan | Central China | *TI* |
| CC57 | Da tou meng | Central China | *IC* | CC132 | Ta gu zhan | Central China | *TI* |
| CC58 | Chi bai gan zhan | Central China | *TI* | CC133 | Mian tiao zhan | Central China | *TI* |
| CC59 | Zeng cheng xiang shan zhan | South China | *TI* | CC134 | Hu guang zhan | Central China | *TI* |
| CC60 | Cang wu shan he zhan | South China | *TI* | CC135 | Jiang an da ye zao | Central China | *TI* |
| CC61 | Da gu zao | South China | *TI* | CC136 | Tie ban zhan | Central China | *TI* |
| CC62 | Jie yang dong liao zhong | South China | *TI* | CC137 | Da gu zao | Central China | *TI* |
| CC63 | Nan xiong ku gua zao | South China | *TI* | CC138 | Ding nan dong zhan | Central China | *TI* |
| CC64 | Chang mang | South China | *TI* | CC139 | Lao wu gu | Central China | *TI* |
| CC65 | Mandi | Celebes | *TI* | CC140 | Xin ban chang ke zi | Central China | *TI* |
| CC66 | Bai gu | South China | *TI* | CC141 | Bai zhan gu | Central China | *TI* |
| CC67 | Hong zao gu | Yunnan-Kweichow Plateau | *TI* | CC142 | Si chuan zhan | Central China | *TI* |
| CC68 | Cang wu shan he zhan | South China | *TI* | CC143 | Gao jiao gui hua | Central China | *TI* |
| CC69 | Zao die zhan gu | Central China | *TI* | CC144 | Chang shu wu wi dao | Central China | *TI* |
| CC70 | Su zhou zhan | Central China | *TI* | CC145 | Da nuo bai dong | Central China | *TI* |
| CC71 | Yang zhan 3 | South China | *TI* | CC146 | Lin chuan da ye zao | South China | *TI* |
| CC72 | Luo ding zhan 1 | South China | *TI* | CC147 | Da bai cao | North China | *TI* |
| CC73 | Gen yin 29 | South China | *TI* | CC148 | Chang ning wu qu nan tou zhan | Central China | *TI* |
| CC74 | Wu ke nuo | South China | *TI* | CC149 | Pi xian da ye zi | Central China | *TI* |
| CC75 | Hua bai ke | South China | *TI* | CC150 | Xi zi zhan | Central China | *TI* |

* *Indica* or *japonica* characteristic were identified by Cheng’s index, i.e. TI, typical *indica* (1-7 score), IC, *indica*_clined (8-13 score), JC, *japonica*_clined (14-17 score), and TJ, typical *japonica* (18-24 score) . Cheng’s index was based on the score of the six phenotypic traits for each variety.

**Table S2. Summary statistics of the 274 SSR markers used in this study**

| **locus** | **Chr No.** | **position**  **(cM)** | **A N** | **GD** | **PIC** |  | **locus** | **Chr No.** | **position**  **(cM)** | **AN** | **GD** | **PIC** |
| --- | --- | --- | --- | --- | --- | --- | --- | --- | --- | --- | --- | --- |
| PSM41 | 1 | 10.9 | 6.0000 | 0.6319 | 0.5944 |  | RM51 | 7 | 0 | 2.0000 | 0.4570 | 0.3526 |
| RM84 | 1 | 26.2 | 5.0000 | 0.4316 | 0.3981 |  | RM427 | 7 | 1.1 | 2.0000 | 0.4146 | 0.3286 |
| RM220 | 1 | 28.4 | 5.0000 | 0.7251 | 0.6805 |  | PSM142 | 7 | 26 | 5.0000 | 0.7519 | 0.7108 |
| RM348-1 | 1 | 28.9 | 2.0000 | 0.4956 | 0.3728 |  | RM180 | 7 | 30.1 | 9.0000 | 0.8128 | 0.7893 |
| RM1 | 1 | 29.7 | 10.0000 | 0.8588 | 0.8426 |  | RM325B | 7 | 33.3 | 2.0000 | 0.0132 | 0.0132 |
| RM283 | 1 | 31.4 | 4.0000 | 0.7162 | 0.6663 |  | RM214 | 7 | 34.7 | 6.0000 | 0.6218 | 0.5837 |
| RM576 | 1 | 51 | 3.0000 | 0.6638 | 0.5897 |  | RM445 | 7 | 39.3 | 2.0000 | 0.3432 | 0.2843 |
| RM259 | 1 | 54.2 | 3.0000 | 0.4783 | 0.4298 |  | RM125 | 7 | 41.7 | 4.0000 | 0.3962 | 0.3368 |
| RM243 | 1 | 57.3 | 5.0000 | 0.5885 | 0.5075 |  | RM432 | 7 | 43.5 | 3.0000 | 0.6589 | 0.5850 |
| RM583 | 1 | 58.9 | 4.0000 | 0.6449 | 0.5713 |  | RM11 | 7 | 47 | 4.0000 | 0.4948 | 0.4546 |
| RM577 | 1 | 61.3 | 2.0000 | 0.4032 | 0.3219 |  | RM346 | 7 | 47 | 4.0000 | 0.5190 | 0.4537 |
| RM81A | 1 | 77.5 | 3.0000 | 0.5704 | 0.5042 |  | RM182 | 7 | 61 | 7.0000 | 0.6560 | 0.5992 |
| RM129 | 1 | 77.5 | 3.0000 | 0.3847 | 0.3203 |  | RM10 | 7 | 63.5 | 3.0000 | 0.4066 | 0.3538 |
| RM562 | 1 | 78.4 | 6.0000 | 0.7738 | 0.7384 |  | RM455 | 7 | 65.7 | 2.0000 | 0.4200 | 0.3318 |
| PSM368 | 1 | 91.2 | 2.0000 | 0.3102 | 0.2661 |  | PSM336 | 7 | 80.5 | 5.0000 | 0.7286 | 0.6806 |
| RM9 | 1 | 92.4 | 6.0000 | 0.8047 | 0.7764 |  | RM234 | 7 | 88.2 | 4.0000 | 0.4387 | 0.3767 |
| RM5 | 1 | 94.9 | 4.0000 | 0.6980 | 0.6429 |  | RM18 | 7 | 90.4 | 2.0000 | 0.4644 | 0.3566 |
| RM306 | 1 | 98.1 | 6.0000 | 0.5593 | 0.5266 |  | RM47 | 7 | 90.4 | 2.0000 | 0.4992 | 0.3746 |
| RM237 | 1 | 115.2 | 3.0000 | 0.5727 | 0.4955 |  | RM429 | 7 | 96.9 | 2.0000 | 0.1128 | 0.1064 |
| RM128 | 1 | 134.8 | 2.0000 | 0.3716 | 0.3026 |  | PSM147 | 7 | 102.3 | 2.0000 | 0.4850 | 0.3674 |
| PSM334 | 1 | 142.4 | 4.0000 | 0.5480 | 0.4452 |  | RM248 | 7 | 116.6 | 10.0000 | 0.8547 | 0.8380 |
| PSM369 | 1 | 170.4 | 3.0000 | 0.6528 | 0.5786 |  | RM408 | 8 | 0 | 3.0000 | 0.5663 | 0.4832 |
| PSM370 | 1 | 176.3 | 2.0000 | 0.4968 | 0.3734 |  | RM337 | 8 | 1.1 | 3.0000 | 0.6175 | 0.5429 |
| RM109 | 2 | 0 | 5.0000 | 0.7206 | 0.6698 |  | PSM392 | 8 | 3.6 | 4.0000 | 0.6182 | 0.5474 |
| RM154 | 2 | 4.8 | 3.0000 | 0.5354 | 0.4588 |  | RM407 | 8 | 5.7 | 2.0000 | 0.4530 | 0.3504 |
| RM110 | 2 | 6.9 | 2.0000 | 0.4992 | 0.3746 |  | RM25 | 8 | 52.2 | 5.0000 | 0.5614 | 0.5166 |
| RM211 | 2 | 14.4 | 4.0000 | 0.6546 | 0.5849 |  | RM547 | 8 | 58.1 | 7.0000 | 0.7728 | 0.7429 |
| RM236 | 2 | 14.4 | 4.0000 | 0.5987 | 0.5306 |  | RM404 | 8 | 60.9 | 4.0000 | 0.6002 | 0.5253 |
| RM279 | 2 | 17.3 | 5.0000 | 0.7174 | 0.6681 |  | RM44 | 8 | 69 | 5.0000 | 0.4406 | 0.4171 |
| RM71 | 2 | 49.8 | 4.0000 | 0.6404 | 0.5716 |  | RM331 | 8 | 69 | 5.0000 | 0.3890 | 0.3395 |
| RM27 | 2 | 66 | 2.0000 | 0.4414 | 0.3440 |  | RM339 | 8 | 72.2 | 5.0000 | 0.6811 | 0.6273 |
| RM29 | 2 | 68.9 | 3.0000 | 0.4727 | 0.3885 |  | RM42 | 8 | 78.4 | 3.0000 | 0.5384 | 0.4445 |
| RM262 | 2 | 70.2 | 4.0000 | 0.7402 | 0.6921 |  | RM342A | 8 | 78.4 | 6.0000 | 0.7429 | 0.7043 |
| RM341 | 2 | 82.7 | 5.0000 | 0.7525 | 0.7133 |  | RM223 | 8 | 80.5 | 5.0000 | 0.7280 | 0.6871 |
| PSM374 | 2 | 83.6 | 2.0000 | 0.3578 | 0.2938 |  | RM284 | 8 | 83.7 | 2.0000 | 0.4712 | 0.3602 |
| PSM122 | 2 | 88.2 | 3.0000 | 0.6383 | 0.5634 |  | RM210 | 8 | 90.3 | 6.0000 | 0.5322 | 0.4838 |
| RM106 | 2 | 123.2 | 2.0000 | 0.4712 | 0.3602 |  | RM419 | 8 | 95.2 | 2.0000 | 0.0526 | 0.0512 |
| RM263 | 2 | 127.5 | 5.0000 | 0.7657 | 0.7270 |  | RM256 | 8 | 101.5 | 2.0000 | 0.0392 | 0.0384 |
| RM450 | 2 | 150.8 | 4.0000 | 0.6135 | 0.5659 |  | RM80 | 8 | 103.7 | 9.0000 | 0.8004 | 0.7747 |
| RM6 | 2 | 154.7 | 3.0000 | 0.6556 | 0.5812 |  | RM149 | 8 | 103.7 | 6.0000 | 0.5680 | 0.4738 |
| RM240 | 2 | 158 | 3.0000 | 0.6335 | 0.5572 |  | RM308 | 8 | 104.8 | 3.0000 | 0.0962 | 0.0940 |
| RM250 | 2 | 170.1 | 5.0000 | 0.6033 | 0.5684 |  | PSM351 | 8 | 119.9 | 3.0000 | 0.4794 | 0.4176 |
| RM530 | 2 | 170.1 | 4.0000 | 0.7339 | 0.6849 |  | RM458 | 8 | 121.8 | 2.0000 | 0.4928 | 0.3714 |
| RM213 | 2 | 186.4 | 4.0000 | 0.6953 | 0.6413 |  | RM447 | 8 | 124.6 | 3.0000 | 0.5848 | 0.5200 |
| RM208 | 2 | 186.4 | 4.0000 | 0.6845 | 0.6258 |  | RM281 | 8 | 128.6 | 5.0000 | 0.6522 | 0.6121 |
| RM207 | 2 | 191.2 | 6.0000 | 0.7572 | 0.7174 |  | RM264 | 8 | 128.6 | 5.0000 | 0.7217 | 0.6775 |
| RM498 | 2 | 194.6 | 2.0000 | 0.4252 | 0.3348 |  | RM296 | 9 | 0 | 2.0000 | 0.4872 | 0.3685 |
| RM138 | 2 | 196.8 | 4.0000 | 0.7359 | 0.6869 |  | RM316 | 9 | 1.8 | 3.0000 | 0.3834 | 0.3276 |
| RM60 | 3 | 0 | 2.0000 | 0.3539 | 0.2913 |  | RM444 | 9 | 3.3 | 6.0000 | 0.5284 | 0.4383 |
| RM104 | 3 | 3.5 | 3.0000 | 0.4676 | 0.4096 |  | RM219 | 9 | 11.7 | 5.0000 | 0.7651 | 0.7275 |
| RM132 | 3 | 7 | 3.0000 | 0.4228 | 0.3707 |  | RM342B | 9 | 14.3 | 4.0000 | 0.6865 | 0.6356 |
| RM22 | 3 | 11 | 4.0000 | 0.6071 | 0.5405 |  | PSM399 | 9 | 21.4 | 3.0000 | 0.5742 | 0.4866 |
| RM348-2 | 3 | 13 | 2.0000 | 0.4772 | 0.3634 |  | PSM157 | 9 | 30.6 | 4.0000 | 0.6566 | 0.6104 |
| RM231 | 3 | 15.7 | 4.0000 | 0.5947 | 0.5229 |  | PSM158 | 9 | 33 | 4.0000 | 0.3985 | 0.3493 |
| RM7 | 3 | 64 | 4.0000 | 0.7252 | 0.6748 |  | PSM160 | 9 | 42.5 | 2.0000 | 0.4509 | 0.3493 |
| RM218 | 3 | 67.8 | 6.0000 | 0.7068 | 0.6556 |  | RM409 | 9 | 45.6 | 2.0000 | 0.0768 | 0.0739 |
| PSM377 | 3 | 69.7 | 4.0000 | 0.7128 | 0.6607 |  | RM566 | 9 | 47.7 | 5.0000 | 0.6918 | 0.6380 |
| RM232 | 3 | 76.7 | 4.0000 | 0.7430 | 0.6952 |  | RM434 | 9 | 57.7 | 4.0000 | 0.6484 | 0.6007 |
| RM251 | 3 | 79.1 | 7.0000 | 0.6074 | 0.5720 |  | PSM337 | 9 | 63 | 2.0000 | 0.3648 | 0.2983 |
| PSM379 | 3 | 80 | 3.0000 | 0.6463 | 0.5732 |  | RM257 | 9 | 66.1 | 7.0000 | 0.7774 | 0.7441 |
| RM16 | 3 | 94.9 | 4.0000 | 0.6619 | 0.5920 |  | PSM338 | 9 | 68.2 | 2.0000 | 0.4911 | 0.3705 |
| PSM128 | 3 | 96.6 | 2.0000 | 0.4048 | 0.3229 |  | RM242 | 9 | 73.3 | 4.0000 | 0.4291 | 0.3575 |
| RM282 | 3 | 100.6 | 2.0000 | 0.4986 | 0.3743 |  | RM553 | 9 | 76.7 | 2.0000 | 0.4252 | 0.3348 |
| RM156 | 3 | 125.7 | 2.0000 | 0.3506 | 0.2891 |  | RM278 | 9 | 77.5 | 4.0000 | 0.7036 | 0.6474 |
| PSM130 | 3 | 130.7 | 2.0000 | 0.3200 | 0.2688 |  | RM201 | 9 | 81.2 | 3.0000 | 0.6239 | 0.5516 |
| RM135 | 3 | 157.3 | 3.0000 | 0.3954 | 0.3427 |  | RM160 | 9 | 82.4 | 8.0000 | 0.4815 | 0.4560 |
| RM293 | 3 | 193.4 | 2.0000 | 0.4608 | 0.3546 |  | PSM340 | 9 | 90.1 | 3.0000 | 0.5407 | 0.4807 |
| RM468 | 3 | 202.3 | 3.0000 | 0.3976 | 0.3474 |  | RM215 | 9 | 99.4 | 2.0000 | 0.2112 | 0.1889 |
| RM571 | 3 | 205.4 | 3.0000 | 0.4852 | 0.3738 |  | RM205 | 9 | 114.7 | 5.0000 | 0.5693 | 0.5237 |
| RM143 | 3 | 207.3 | 3.0000 | 0.6539 | 0.5797 |  | RM222 | 10 | 11.3 | 3.0000 | 0.2063 | 0.1945 |
| RM130 | 3 | 208.2 | 2.0000 | 0.3648 | 0.2983 |  | RM244 | 10 | 15 | 2.0000 | 0.3848 | 0.3108 |
| RM565 | 3 | 215.5 | 2.0000 | 0.3645 | 0.2981 |  | RM216 | 10 | 17.6 | 5.0000 | 0.7500 | 0.7082 |
| RM307 | 4 | 0 | 3.0000 | 0.6516 | 0.5765 |  | PSM163 | 10 | 21.8 | 2.0000 | 0.3036 | 0.2575 |
| RM401 | 4 | 8.5 | 3.0000 | 0.5464 | 0.4855 |  | PSM164 | 10 | 24.2 | 2.0000 | 0.0644 | 0.0624 |
| RM551 | 4 | 8.5 | 4.0000 | 0.6026 | 0.5592 |  | RM239 | 10 | 25.2 | 2.0000 | 0.4090 | 0.3253 |
| RM335 | 4 | 21.5 | 7.0000 | 0.6846 | 0.6554 |  | RM311 | 10 | 25.2 | 5.0000 | 0.7690 | 0.7318 |
| RM518 | 4 | 25.5 | 3.0000 | 0.6644 | 0.5903 |  | PSM166 | 10 | 32.1 | 6.0000 | 0.8135 | 0.7868 |
| RM471 | 4 | 53.8 | 3.0000 | 0.6016 | 0.5237 |  | PSM167 | 10 | 55.6 | 4.0000 | 0.5336 | 0.4921 |
| RM564 | 4 | 73.1 | 3.0000 | 0.6522 | 0.5788 |  | RM184 | 10 | 58.3 | 2.0000 | 0.4872 | 0.3685 |
| RM119 | 4 | 76.1 | 4.0000 | 0.4947 | 0.4332 |  | RM271 | 10 | 59.4 | 5.0000 | 0.5902 | 0.5243 |
| RM273 | 4 | 94.4 | 3.0000 | 0.5255 | 0.4127 |  | PSM169 | 10 | 61.4 | 2.0000 | 0.4892 | 0.3696 |
| RM252 | 4 | 99 | 5.0000 | 0.6750 | 0.6168 |  | PSM170 | 10 | 68.6 | 2.0000 | 0.3682 | 0.3004 |
| RM241 | 4 | 106.2 | 4.0000 | 0.7331 | 0.6845 |  | RM269 | 10 | 69.6 | 4.0000 | 0.6891 | 0.6314 |
| PSM102 | 4 | 108.2 | 4.0000 | 0.7062 | 0.6537 |  | RM258 | 10 | 70.8 | 4.0000 | 0.6015 | 0.5375 |
| RM451 | 4 | 115.5 | 2.0000 | 0.4422 | 0.3444 |  | RM304 | 10 | 73 | 6.0000 | 0.7842 | 0.7509 |
| RM303 | 4 | 116.9 | 5.0000 | 0.4532 | 0.4142 |  | RM171 | 10 | 73 | 2.0000 | 0.4090 | 0.3253 |
| RM317 | 4 | 118.3 | 3.0000 | 0.4316 | 0.3794 |  | RM228 | 10 | 96.3 | 12.0000 | 0.8227 | 0.8023 |
| PSM382 | 4 | 122.9 | 2.0000 | 0.3783 | 0.3068 |  | RM484 | 10 | 97.3 | 2.0000 | 0.1906 | 0.1724 |
| RM255 | 4 | 135.4 | 4.0000 | 0.4062 | 0.3603 |  | RM147 | 10 | 99.8 | 2.0000 | 0.4850 | 0.3674 |
| RM348 | 4 | 137.9 | 2.0000 | 0.3648 | 0.2983 |  | RM333 | 10 | 110.4 | 7.0000 | 0.7355 | 0.6939 |
| RM349 | 4 | 146.8 | 3.0000 | 0.5491 | 0.4772 |  | RM496 | 10 | 113 | 2.0000 | 0.3432 | 0.2843 |
| RM127 | 4 | 150.1 | 3.0000 | 0.4008 | 0.3256 |  | RM590 | 10 | 117.2 | 3.0000 | 0.3796 | 0.3170 |
| RM280 | 4 | 152.3 | 3.0000 | 0.5108 | 0.4560 |  | RM591 | 10 | 118.3 | 8.0000 | 0.5627 | 0.5270 |
| RM559 | 4 | 155.8 | 2.0000 | 0.4352 | 0.3405 |  | RM286 | 11 | 0 | 7.0000 | 0.7124 | 0.6705 |
| RM122 | 5 | 0 | 4.0000 | 0.4026 | 0.3794 |  | PSM171 | 11 | 4.8 | 2.0000 | 0.3279 | 0.2741 |
| RM153 | 5 | 3 | 3.0000 | 0.5032 | 0.4364 |  | PSM172 | 11 | 7.6 | 2.0000 | 0.4466 | 0.3469 |
| PSM341 | 5 | 6.5 | 3.0000 | 0.5876 | 0.5156 |  | RM167 | 11 | 20.3 | 4.0000 | 0.4573 | 0.4225 |
| PSM8 | 5 | 13 | 4.0000 | 0.6225 | 0.5498 |  | PSM410 | 11 | 35.6 | 3.0000 | 0.4451 | 0.3975 |
| PSM60 | 5 | 13 | 4.0000 | 0.6159 | 0.5596 |  | PSM411 | 11 | 49.6 | 3.0000 | 0.5810 | 0.5055 |
| PSM202 | 5 | 14.1 | 3.0000 | 0.4163 | 0.3400 |  | RM202 | 11 | 54 | 3.0000 | 0.5749 | 0.5096 |
| RM413 | 5 | 26.7 | 5.0000 | 0.6213 | 0.5770 |  | RM536 | 11 | 55.1 | 4.0000 | 0.6858 | 0.6333 |
| RM267 | 5 | 28.6 | 4.0000 | 0.4620 | 0.3989 |  | RM287 | 11 | 68.6 | 6.0000 | 0.6953 | 0.6510 |
| RM405 | 5 | 28.6 | 5.0000 | 0.6222 | 0.5652 |  | RM209 | 11 | 73.9 | 5.0000 | 0.6604 | 0.6022 |
| RM548 | 5 | 28.6 | 2.0000 | 0.3944 | 0.3167 |  | PSM416 | 11 | 74.9 | 2.0000 | 0.4608 | 0.3546 |
| RM249 | 5 | 54.3 | 7.0000 | 0.4787 | 0.4370 |  | RM229 | 11 | 77.8 | 5.0000 | 0.7360 | 0.6887 |
| RM169 | 5 | 57.9 | 8.0000 | 0.8393 | 0.8187 |  | RM457 | 11 | 83 | 2.0000 | 0.4968 | 0.3734 |
| RM516 | 5 | 59.6 | 2.0000 | 0.1010 | 0.0959 |  | PSM365 | 11 | 84.6 | 6.0000 | 0.7857 | 0.7545 |
| RM509 | 5 | 65.8 | 3.0000 | 0.3111 | 0.2882 |  | RM21 | 11 | 85.7 | 6.0000 | 0.6186 | 0.5824 |
| RM146 | 5 | 78.7 | 2.0000 | 0.4488 | 0.3481 |  | PSM366 | 11 | 89 | 4.0000 | 0.5500 | 0.4477 |
| RM164 | 5 | 79.4 | 4.0000 | 0.6215 | 0.5763 |  | PSM417 | 11 | 97.3 | 5.0000 | 0.7033 | 0.6485 |
| RM31 | 5 | 118.8 | 4.0000 | 0.7349 | 0.6862 |  | RM206 | 11 | 102.9 | 9.0000 | 0.5073 | 0.4886 |
| PSM386 | 5 | 123 | 2.0000 | 0.0890 | 0.0850 |  | PSM418 | 11 | 104.9 | 6.0000 | 0.7546 | 0.7175 |
| RM274 | 5 | 126.6 | 2.0000 | 0.4090 | 0.3253 |  | RM254 | 11 | 110 | 3.0000 | 0.4068 | 0.3293 |
| RM538 | 5 | 132.7 | 2.0000 | 0.4978 | 0.3739 |  | PSM176 | 11 | 117.9 | 3.0000 | 0.4282 | 0.3815 |
| RM334 | 5 | 141.8 | 7.0000 | 0.5857 | 0.5481 |  | RM224 | 11 | 120.1 | 5.0000 | 0.6621 | 0.6103 |
| RM469 | 6 | 2.2 | 3.0000 | 0.6593 | 0.5853 |  | RM144 | 11 | 123.2 | 5.0000 | 0.6143 | 0.5540 |
| RM170 | 6 | 2.2 | 5.0000 | 0.5832 | 0.5258 |  | RM20A | 12 | 3.2 | 2.0000 | 0.3648 | 0.2983 |
| RM589 | 6 | 3.2 | 5.0000 | 0.6827 | 0.6177 |  | RM4A | 12 | 5.2 | 3.0000 | 0.6374 | 0.5632 |
| RM510 | 6 | 20.8 | 2.0000 | 0.4002 | 0.3201 |  | PSM183 | 12 | 12.2 | 3.0000 | 0.6562 | 0.5820 |
| RM204 | 6 | 25.1 | 10.0000 | 0.7537 | 0.7297 |  | RM19 | 12 | 20.9 | 2.0000 | 0.3911 | 0.3146 |
| RM217 | 6 | 26.2 | 5.0000 | 0.7761 | 0.7417 |  | PSM184 | 12 | 26 | 4.0000 | 0.5528 | 0.4877 |
| RM225 | 6 | 26.2 | 4.0000 | 0.6719 | 0.6147 |  | PSM419 | 12 | 30 | 5.0000 | 0.3674 | 0.3475 |
| RM584 | 6 | 26.2 | 3.0000 | 0.6282 | 0.5554 |  | RM247 | 12 | 32.3 | 8.0000 | 0.5462 | 0.5264 |
| RM111 | 6 | 35.3 | 2.0000 | 0.4090 | 0.3253 |  | PSM420 | 12 | 42.7 | 3.0000 | 0.5852 | 0.4982 |
| RM253 | 6 | 37 | 5.0000 | 0.7147 | 0.6615 |  | RM179 | 12 | 46.8 | 2.0000 | 0.2351 | 0.2075 |
| RM402 | 6 | 40.3 | 3.0000 | 0.5848 | 0.4948 |  | RM101 | 12 | 49.5 | 3.0000 | 0.4344 | 0.3457 |
| RM276 | 6 | 40.3 | 11.0000 | 0.6756 | 0.6451 |  | PSM421 | 12 | 55.9 | 3.0000 | 0.6500 | 0.5746 |
| RM557 | 6 | 41.9 | 2.0000 | 0.4967 | 0.3734 |  | RM277 | 12 | 57.2 | 2.0000 | 0.3972 | 0.3183 |
| RM549 | 6 | 42.7 | 3.0000 | 0.5930 | 0.5225 |  | RM519 | 12 | 62.6 | 3.0000 | 0.6479 | 0.5722 |
| RM539 | 6 | 45.1 | 4.0000 | 0.7428 | 0.6950 |  | RM309 | 12 | 74.5 | 3.0000 | 0.1384 | 0.1334 |
| RM136 | 6 | 51.2 | 3.0000 | 0.5039 | 0.3835 |  | RM463 | 12 | 75.5 | 2.0000 | 0.4252 | 0.3348 |
| RM3 | 6 | 74.3 | 6.0000 | 0.7678 | 0.7323 |  | PSM188 | 12 | 86.5 | 3.0000 | 0.6563 | 0.5825 |
| RM541 | 6 | 75.5 | 7.0000 | 0.8038 | 0.7753 |  | RM270 | 12 | 91.3 | 4.0000 | 0.3331 | 0.3118 |
| PSM138 | 6 | 79 | 3.0000 | 0.6115 | 0.5376 |  | RM235 | 12 | 91.3 | 7.0000 | 0.8146 | 0.7900 |
| RM162 | 6 | 108.3 | 5.0000 | 0.6596 | 0.6013 |  | PSM190 | 12 | 95.1 | 8.0000 | 0.8011 | 0.7760 |
| RM340 | 6 | 133.5 | 5.0000 | 0.6920 | 0.6561 |  | PSM191 | 12 | 99.7 | 2.0000 | 0.1010 | 0.0959 |
| RM400 | 6 | 134.5 | 9.0000 | 0.5338 | 0.5066 |  | RM17 | 12 | 109.1 | 3.0000 | 0.4367 | 0.3703 |
| **Total alleles**  **Mean** |  |  |  |  |  |  |  |  |  | **1063**  **3.8796** | **0.5436** | **0.4831** |

**Table S3.** Allele frequency of the 55 significant markers in three panels

| No. | | Significant markers | Allele(bp) | Allele frequency(%) | | |
| --- | --- | --- | --- | --- | --- | --- |
|  |  |  |  | Panel 1 | Panel 2 | Panel 3 |
| 1 | RM81A | | 105 | 0 | 2.6 | 4.2 |
|  |  | | 110 | 16.0 | 44.6 | 21.0 |
|  |  | | 123 | 58.0 | 37.4 | 49.6 |
|  |  | | 131 | 26.0 | 13.3 | 25.2 |
|  |  | | Missing | 0 | 2.1 | 0 |
| 2 | RM237 | | 105 | 0 | 5.1 | 1.7 |
|  |  | | 115 | 12.0 | 16.9 | 11.8 |
|  |  | | 124 | 55.3 | 43.1 | 47.0 |
|  |  | | 130 | 32.7 | 34.4 | 39.5 |
|  |  | | Missing | 0 | 0.5 | 0 |
| 3 | RM341 | | 129 | 35.3 | 29.2 | 18.5 |
|  |  | | 133 | 12.0 | 19.0 | 10.9 |
|  |  | | 141 | 15.3 | 10.3 | 11.8 |
|  |  | | 151 | 27.3 | 36.4 | 48.7 |
|  |  | | 153 | 10.0 | 4.1 | 9.3 |
|  |  | | Missing | 0 | 0 | 0.8 |
| 4 | RM530 | | 130 | 16.7 | 14.9 | 11.8 |
|  |  | | 136 | 20.6 | 18.5 | 16.8 |
|  |  | | 141 | 0 | 1.0 | 0 |
|  |  | | 144 | 28.0 | 29.7 | 36.1 |
|  |  | | 155 | 32.7 | 35.9 | 35.3 |
|  |  | | Missing | 2.0 | 0 | 0 |
| 5 | RM138 | | 130 | 16.0 | 53.3 | 60.5 |
|  |  | | 136 | 20.7 | 13.8 | 19.3 |
|  |  | | 144 | 28.0 | 21.6 | 4.2 |
|  |  | | 155 | 30.0 | 11.3 | 16.0 |
|  |  | | Missing | 5.3 | 0 | 0 |
| 6 | RM208 | | 152 | 36.0 | 46.2 | 23.5 |
|  |  | | 156 | 15.3 | 20.0 | 15.2 |
|  |  | | 160 | 38.7 | 21.5 | 21.8 |
|  |  | | 167 | 9.3 | 12.3 | 39.5 |
|  |  | | Missing | 0.7 | 0 | 0 |
| 7 | PSM374 | | 213 | 23.3 | 30.8 | 26.0 |
|  |  | | 242 | 76.7 | 69.2 | 74.0 |
| 8 | PSM130 | | 202 | 80.0 | 67.2 | 87.4 |
|  |  | | 216 | 20.0 | 32.8 | 12.6 |
| 9 | RM156 | | 139 | 77.3 | 67.2 | 86.6 |
|  |  | | 144 | 22.7 | 32.8 | 13.4 |
| 10 | PSM128 | | 142 | 71.3 | 77.4 | 81.5 |
|  |  | | 148 | 28.0 | 22.6 | 18.5 |
|  |  | | Missing | 0.7 | 0 | 0 |
| 11 | RM571 | | 169 | 39.3 | 44.1 | 40.3 |
|  |  | | 175 | 60.0 | 45.1 | 43.7 |
|  |  | | 190 | 0.7 | 10.8 | 16.0 |
| 12 | RM7 | | 139 | 14.0 | 21.0 | 17.6 |
|  |  | | 152 | 35.3 | 26.2 | 24.4 |
|  |  | | 155 | 27.3 | 40.0 | 49.6 |
|  |  | | 160 | 22.0 | 12.8 | 8.4 |
|  |  | | Missing | 13.4 | 0 | 0 |
| 13 | RM127 | | 179 | 72.7 | 41.0 | 72.3 |
|  |  | | 191 | 26.6 | 35.4 | 16.8 |
|  |  | | 200 | 0.7 | 23.6 | 10.9 |
| 14 | RM559 | | 145 | 68.0 | 70.3 | 75.6 |
|  |  | | 152 | 32.0 | 28.7 | 21.9 |
|  |  | | 180 | 0 | 1.0 | 2.5 |
| 15 | RM348 | | 124 | 76.0 | 68.2 | 85.7 |
|  |  | | 138 | 24.0 | 31.8 | 14.3 |
| 16 | RM252 | | 163 | 43.3 | 24.1 | 6.7 |
|  |  | | 166 | 4.0 | 16.9 | 5.9 |
|  |  | | 179 | 16.7 | 21.0 | 22.7 |
|  |  | | 198 | 32.7 | 19.0 | 37.8 |
|  |  | | 201 | 3.3 | 14.9 | 23.5 |
|  |  | | 215 | 0 | 4.1 | 3.4 |
| 17 | RM153 | | 155 | 0 | 2.6 | 1.7 |
|  |  | | 162 | 8.7 | 36.9 | 14.3 |
|  |  | | 172 | 64.7 | 50.2 | 54.6 |
|  |  | | 175 | 26.6 | 10.3 | 29.4 |
| 18 | RM538 | | 249 | 46.7 | 44.6 | 50.4 |
|  |  | | 255 | 53.3 | 55.4 | 49.6 |
| 19 | RM469 | | 83 | 38.0 | 28.7 | 36.1 |
|  |  | | 88 | 28.0 | 13.3 | 8.4 |
|  |  | | 94 | 33.3 | 46.7 | 47.1 |
|  |  | | 105 | 0 | 11.3 | 8.4 |
|  |  | | Missing | 0.7 | 0 | 0 |
| 20 | RM204 | | 98 | 42.0 | 23.1 | 13.4 |
|  |  | | 100 | 15.4 | 12.3 | 27.7 |
|  |  | | 107 | 13.3 | 16.4 | 15.1 |
|  |  | | 116 | 5.3 | 10.2 | 7.6 |
|  |  | | 127 | 4.0 | 13.8 | 10.9 |
|  |  | | 139 | 2.0 | 8.7 | 4.2 |
|  |  | | 142 | 6.0 | 8.3 | 2.5 |
|  |  | | 150 | 6.0 | 1.5 | 7.6 |
|  |  | | 153 | 2.0 | 3.6 | 5.9 |
|  |  | | 162 | 0.7 | 2.1 | 5.0 |
|  |  | | Missing | 3.3 | 0 | 0 |
| 21 | RM225 | | 108 | 30.7 | 28.7 | 23.5 |
|  |  | | 116 | 9.3 | 23.6 | 18.5 |
|  |  | | 120 | 14.6 | 11.8 | 19.3 |
|  |  | | 130 | 44.7 | 35.9 | 38.7 |
|  |  | | Missing | 0.7 | 0 | 0 |
| 22 | RM276 | | 77 | 0.7 | 6.2 | 11.8 |
|  |  | | 90 | 0.7 | 3.6 | 16.0 |
|  |  | | 92 | 1.3 | 0 | 0 |
|  |  | | 100 | 5.3 | 13.3 | 6.7 |
|  |  | | 102 | 4.0 | 0 | 0 |
|  |  | | 107 | 0.7 | 0 | 0 |
|  |  | | 113 | 8.0 | 4.6 | 17.6 |
|  |  | | 120 | 20.0 | 27.2 | 15.1 |
|  |  | | 134 | 2.6 | 21.5 | 16.0 |
|  |  | | 137 | 52.0 | 0 | 0 |
|  |  | | 139 | 4.7 | 14.4 | 9.2 |
|  |  | | 145 | 0 | 9.2 | 7.6 |
| 23 | RM557 | | 198 | 0 | 4.1 | 5.9 |
|  |  | | 201 | 53.4 | 62.6 | 76.5 |
|  |  | | 206 | 45.3 | 33.3 | 17.6 |
|  |  | | Missing | 13.3 | 0 | 0 |
| 24 | RM584 | | 154 | 30.7 | 26.7 | 31.1 |
|  |  | | 164 | 20.7 | 27.7 | 17.6 |
|  |  | | 171 | 48.6 | 45.6 | 51.3 |
| 25 | RM18 | | 128 | 63.3 | 35.9 | 25.3 |
|  |  | | 135 | 36.7 | 53.8 | 52.9 |
|  |  | | 145 | 0 | 51.3 | 21.8 |
| 26 | RM182 | | 284 | 30.6 | 7.7 | 0.8 |
|  |  | | 295 | 6.7 | 7.7 | 6.7 |
|  |  | | 308 | 10.7 | 9.7 | 20.2 |
|  |  | | 320 | 47.3 | 49.7 | 47.1 |
|  |  | | 336 | 13.3 | 4.2 | 16.8 |
|  |  | | 348 | 0.7 | 11.8 | 4.2 |
|  |  | | 355 | 0.7 | 9.2 | 4.2 |
|  |  | | Missing | 2.0 | 0 | 0 |
| 27 | RM339 | | 130 | 27.3 | 17.9 | 5.9 |
|  |  | | 136 | 44.1 | 41.6 | 30.2 |
|  |  | | 142 | 18.0 | 16.9 | 46.2 |
|  |  | | 145 | 7.3 | 20.5 | 14.3 |
|  |  | | 161 | 1.3 | 3.1 | 3.4 |
|  |  | | Missing | 2.0 | 0 | 0 |
| 28 | RM223 | | 124 | 14.0 | 15.4 | 6.7 |
|  |  | | 132 | 23.4 | 16.9 | 19.3 |
|  |  | | 137 | 15.3 | 31.8 | 26.1 |
|  |  | | 142 | 41.3 | 26.7 | 42.0 |
|  |  | | 148 | 6.0 | 9.2 | 5.9 |
| 29 | RM447 | | 100 | 56.7 | 44.1 | 39.5 |
|  |  | | 109 | 20.7 | 35.4 | 36.1 |
|  |  | | 117 | 22.6 | 20.5 | 24.4 |
| 30 | RM407 | | 153 | 34.0 | 42.6 | 31.1 |
|  |  | | 160 | 66.0 | 39.5 | 26.0 |
|  |  | | 165 | 0 | 16.9 | 42.9 |
|  |  | | Missing | 0 | 1.0 | 0 |
| 31 | RM219 | | 180 | 26.6 | 10.3 | 4.2 |
|  |  | | 184 | 14.7 | 6.7 | 19.3 |
|  |  | | 198 | 32.7 | 23.6 | 5.9 |
|  |  | | 200 | 10.0 | 26.2 | 24.4 |
|  |  | | 211 | 16.0 | 28.1 | 38.6 |
|  |  | | 220 | 0 | 5.1 | 7.6 |
| 32 | PSM158 | | 144 | 20.7 | 15.9 | 22.7 |
|  |  | | 152 | 74.7 | 55.9 | 38.7 |
|  |  | | 170 | 1.3 | 26.2 | 35.3 |
|  |  | | 177 | 3.3 | 2.0 | 3.3 |
| 33 | PSM340 | | 154 | 14.7 | 10.3 | 15.2 |
|  |  | | 160 | 62.7 | 64.6 | 65.5 |
|  |  | | 165 | 22.6 | 8.2 | 6.7 |
|  |  | | 170 | 0 | 16.9 | 12.6 |
| 34 | PSM167 | | 188 | 64.7 | 15.9 | 37.0 |
|  |  | | 194 | 18.0 | 62.6 | 46.2 |
|  |  | | 206 | 6.7 | 13.8 | 8.4 |
|  |  | | 215 | 10.6 | 7.7 | 8.4 |
| 35 | RM147 | | 90 | 58.7 | 45.6 | 47.1 |
|  |  | | 96 | 41.3 | 54.4 | 52.9 |
| 36 | RM590 | | 110 | 0 | 1.0 | 0.8 |
|  |  | | 121 | 23.3 | 19.0 | 21.8 |
|  |  | | 130 | 74.7 | 40.0 | 37.9 |
|  |  | | 132 | 1.3 | 40.0 | 39.5 |
|  |  | | Missing | 0.7 | 0 | 0 |
| 37 | RM228 | | 89 | 13.3 | 17.4 | 0.8 |
|  |  | | 100 | 14.0 | 27.7 | 12.6 |
|  |  | | 109 | 18.7 | 15.4 | 1.7 |
|  |  | | 112 | 6.0 | 0 | 0 |
|  |  | | 117 | 0.7 | 8.7 | 4.3 |
|  |  | | 120 | 4.0 | 0 | 0 |
|  |  | | 122 | 1.3 | 3.6 | 16.0 |
|  |  | | 125 | 4.0 | 0 | 0 |
|  |  | | 133 | 30.7 | 18.5 | 21.0 |
|  |  | | 136 | 3.3 | 0 | 0 |
|  |  | | 145 | 2.0 | 7.2 | 13.4 |
|  |  | | 150 | 1.3 | 0.5 | 13.4 |
|  |  | | 160 | 0 | 0.5 | 11.8 |
|  |  | | 170 | 0 | 0.5 | 5.0 |
|  |  | | Missing | 0.7 | 0 | 0 |
| 38 | RM222 | | 195 | 8.0 | 18.5 | 14.3 |
|  |  | | 198 | 88.7 | 70.2 | 51.3 |
|  |  | | 205 | 3.3 | 11.3 | 34.4 |
| 39 | PSM170 | | 213 | 74.7 | 74.4 | 91.6 |
|  |  | | 217 | 24.0 | 25.6 | 7.6 |
|  |  | | Missing | 1.3 | 0 | 0.8 |
| 40 | RM239 | | 165 | 71.3 | 43.6 | 90.8 |
|  |  | | 172 | 28.7 | 56.4 | 9.2 |
| 41 | RM311 | | 143 | 13.3 | 16.4 | 12.6 |
|  |  | | 145 | 32.0 | 43.1 | 35.3 |
|  |  | | 147 | 24.7 | 29.2 | 42.9 |
|  |  | | 153 | 20.0 | 11.3 | 9.2 |
|  |  | | 157 | 10.0 | 0 | 0 |
| 42 | RM224 | | 113 | 14.7 | 22.1 | 9.2 |
|  |  | | 116 | 24.0 | 10.8 | 5.0 |
|  |  | | 120 | 7.3 | 17.9 | 41.2 |
|  |  | | 128 | 45.4 | 11.3 | 25.2 |
|  |  | | 137 | 1.3 | 17.5 | 12.7 |
|  |  | | 150 | 0 | 19.4 | 6.7 |
|  |  | | Missing | 7.3 | 0 | 0 |
| 43 | RM21 | | 125 | 57.3 | 47.7 | 79.8 |
|  |  | | 127 | 5.3 | 10.8 | 5.0 |
|  |  | | 131 | 11.4 | 14.9 | 5.9 |
|  |  | | 135 | 18.0 | 14.4 | 5.9 |
|  |  | | 144 | 6.7 | 7.7 | 2.6 |
|  |  | | 149 | 1.3 | 4.5 | 0.8 |
| 44 | PSM171 | | 190 | 0 | 19.0 | 0 |
|  |  | | 200 | 79.3 | 63.6 | 83.2 |
|  |  | | 210 | 20.7 | 10.8 | 16.8 |
|  |  | | 225 | 0 | 5.6 | 0 |
|  |  | | Missing | 0 | 1.0 | 0 |
| 45 | RM144 | | 215 | 53.4 | 50.8 | 57.1 |
|  |  | | 228 | 29.3 | 18.5 | 22.7 |
|  |  | | 245 | 11.3 | 16.4 | 10.1 |
|  |  | | 260 | 1.3 | 9.7 | 6.7 |
|  |  | | 280 | 4.7 | 4.6 | 3.4 |
| 46 | RM167 | | 121 | 71.4 | 66.2 | 83.2 |
|  |  | | 135 | 16.0 | 28.2 | 13.4 |
|  |  | | 137 | 7.3 | 0 | 0 |
|  |  | | 141 | 5.3 | 5.6 | 3.4 |
| 47 | RM287 | | 80 | 2.0 | 4.1 | 6.7 |
|  |  | | 100 | 44.7 | 54.9 | 22.8 |
|  |  | | 105 | 20.0 | 21.0 | 31.9 |
|  |  | | 110 | 11.3 | 13.8 | 15.1 |
|  |  | | 114 | 17.3 | 6.2 | 23.5 |
|  |  | | Missing | 4.7 | 0 | 0 |
| 48 | PSM172 | | 145 | 66.0 | 62.6 | 84.0 |
|  |  | | 151 | 34.0 | 37.4 | 16.0 |
| 49 | RM206 | | 123 | 7.3 | 7.2 | 40.3 |
|  |  | | 125 | 4.0 | 0 | 0 |
|  |  | | 130 | 57.3 | 28.7 | 14.3 |
|  |  | | 143 | 6.7 | 13.8 | 19.4 |
|  |  | | 145 | 1.3 | 0 | 0 |
|  |  | | 160 | 1.3 | 28.2 | 23.5 |
|  |  | | 162 | 4.0 | 0 | 0 |
|  |  | | 167 | 0.7 | 0 | 0 |
|  |  | | 175 | 0.7 | 15.9 | 2.5 |
|  |  | | 180 | 0 | 6.2 | 0 |
|  |  | | Missing | 16.7 | 0 | 0 |
| 50 | PSM184 | | 205 | 60.0 | 59.0 | 80.7 |
|  |  | | 215 | 26.7 | 13.3 | 5.9 |
|  |  | | 222 | 12.6 | 18.5 | 10.9 |
|  |  | | 240 | 0.7 | 9.2 | 2.5 |
| 51 | RM4A | | 128 | 32.7 | 37.9 | 42.0 |
|  |  | | 133 | 21.3 | 62.1 | 58.0 |
|  |  | | 137 | 45.3 | 0 | 0 |
|  |  | | Missing | 0.7 | 0 | 0 |
| 52 | RM277 | | 111 | 27.3 | 33.3 | 18.5 |
|  |  | | 117 | 72.7 | 64.6 | 72.3 |
|  |  | | 121 | 0 | 2.1 | 9.2 |
| 53 | PSM191 | | 171 | 94.7 | 92.3 | 98.3 |
|  |  | | 175 | 5.3 | 7.7 | 1.7 |
| 54 | RM19 | | 183 | 26.7 | 40.0 | 37.0 |
|  |  | | 190 | 0 | 1.5 | 5.0 |
|  |  | | 198 | 0 | 1.5 | 4.2 |
|  |  | | 203 | 73.3 | 57.0 | 27.7 |
|  |  | | 208 | 0 | 0 | 26.1 |
| 55 | RM235 | | 91 | 12.7 | 35.4 | 17.6 |
|  |  | | 108 | 15.3 | 19.5 | 20.2 |
|  |  | | 115 | 9.3 | 12.3 | 15.1 |
|  |  | | 117 | 10.7 | 0 | 0 |
|  |  | | 121 | 17.3 | 22.1 | 30.3 |
|  |  | | 123 | 28.0 | 0 | 0 |
|  |  | | 125 | 2.0 | 10.7 | 16.8 |
|  |  | | Missing | 4.7 | 0 | 0 |


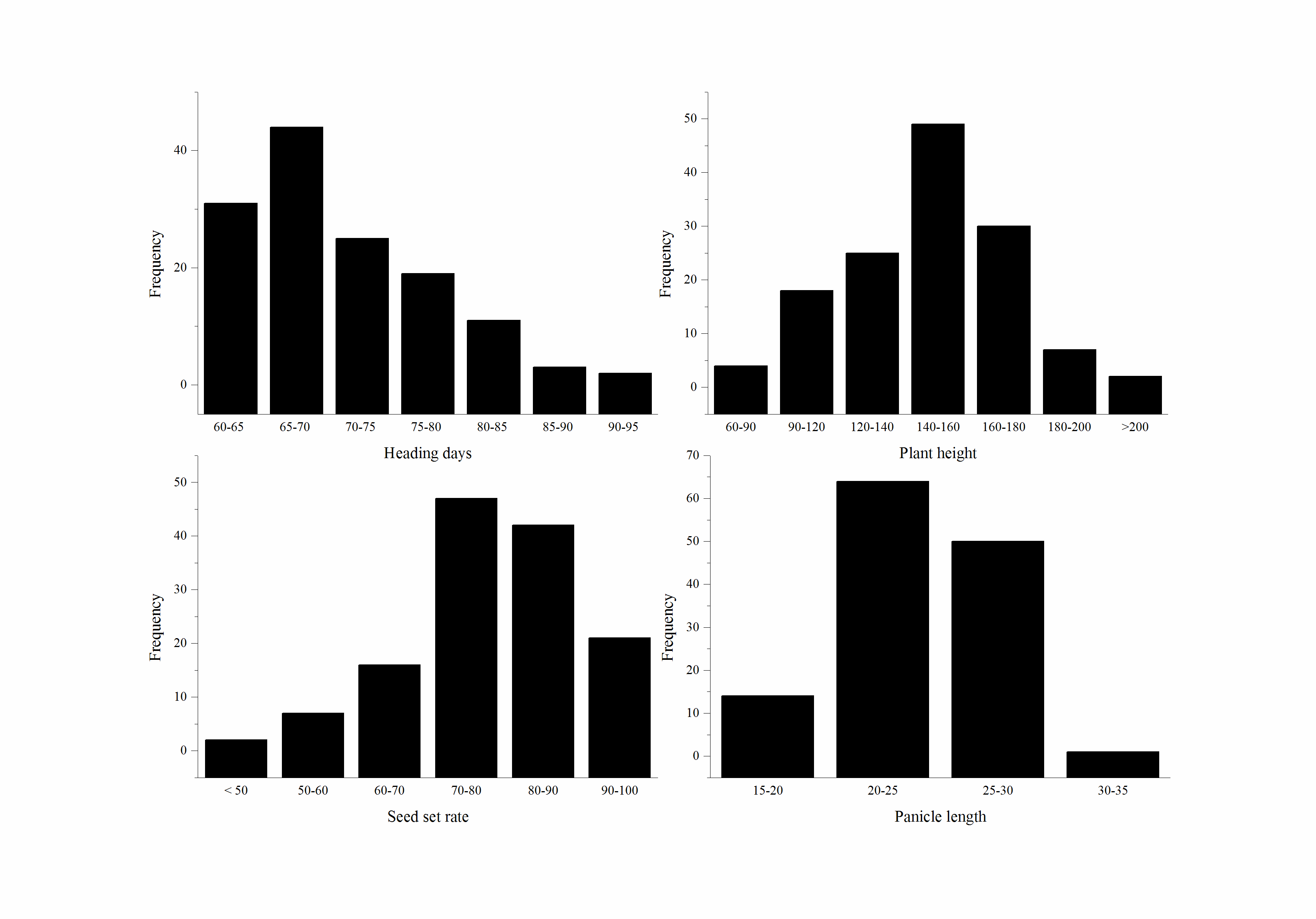


**Figure S1. Frequency distribution of heading days, plant height, seed set rate and panicle length in Panel 1 in 2008.** The height of black bar represents the number of varieties in different range of traits


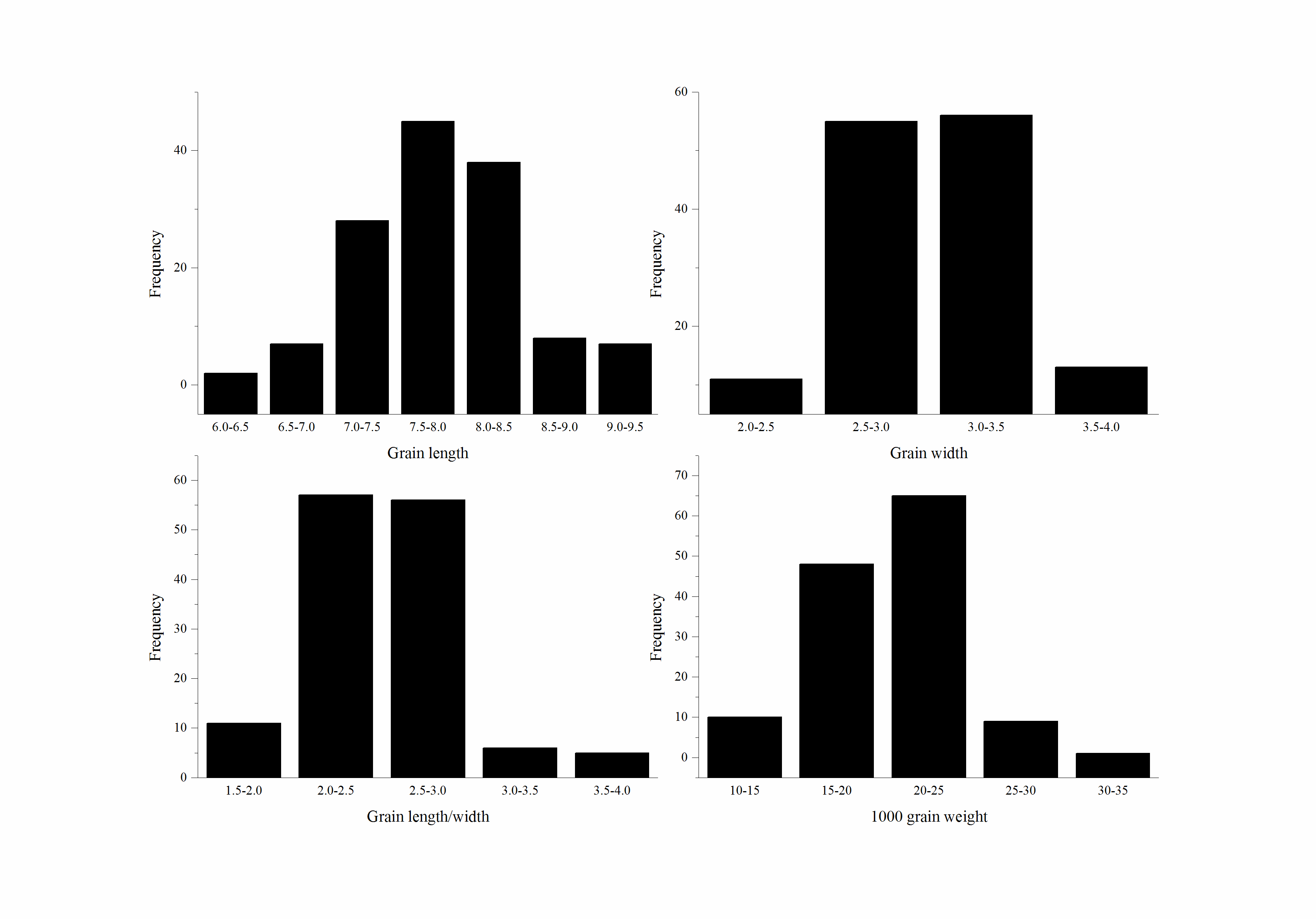


**Figure S2. Frequency distribution of grain length, grain width, grain length/width and 1000 grain weight in Panel 1 in 2008.** The height of black bar represents the number of varieties in different range of traits.


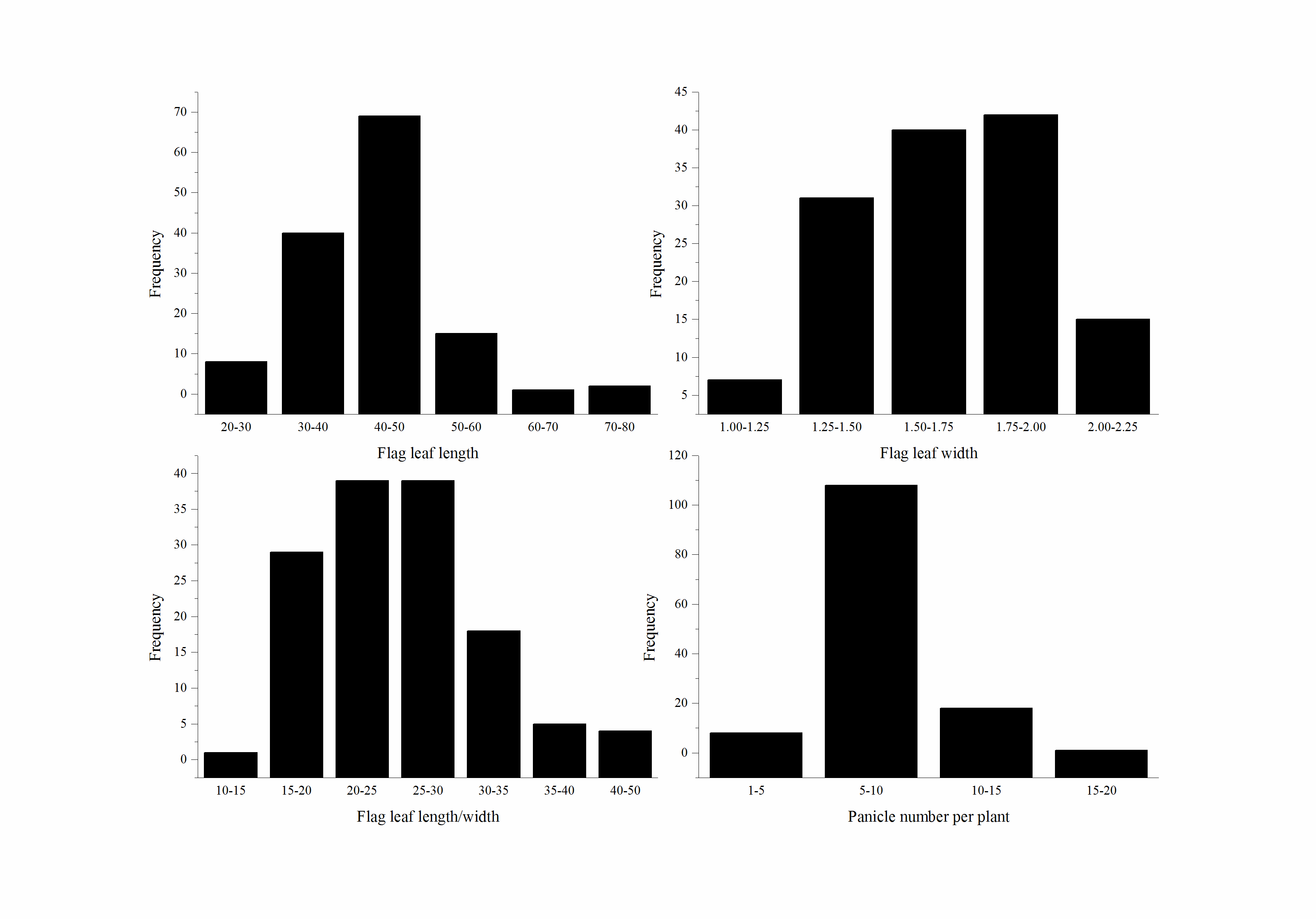


**Figure S3. Frequency distribution of flag leaf length, flag leaf width, flag leaf length/width and panicle number per plant in Panel 1 in 2008.** The height of black bar represents the number of varieties in different range of traits.


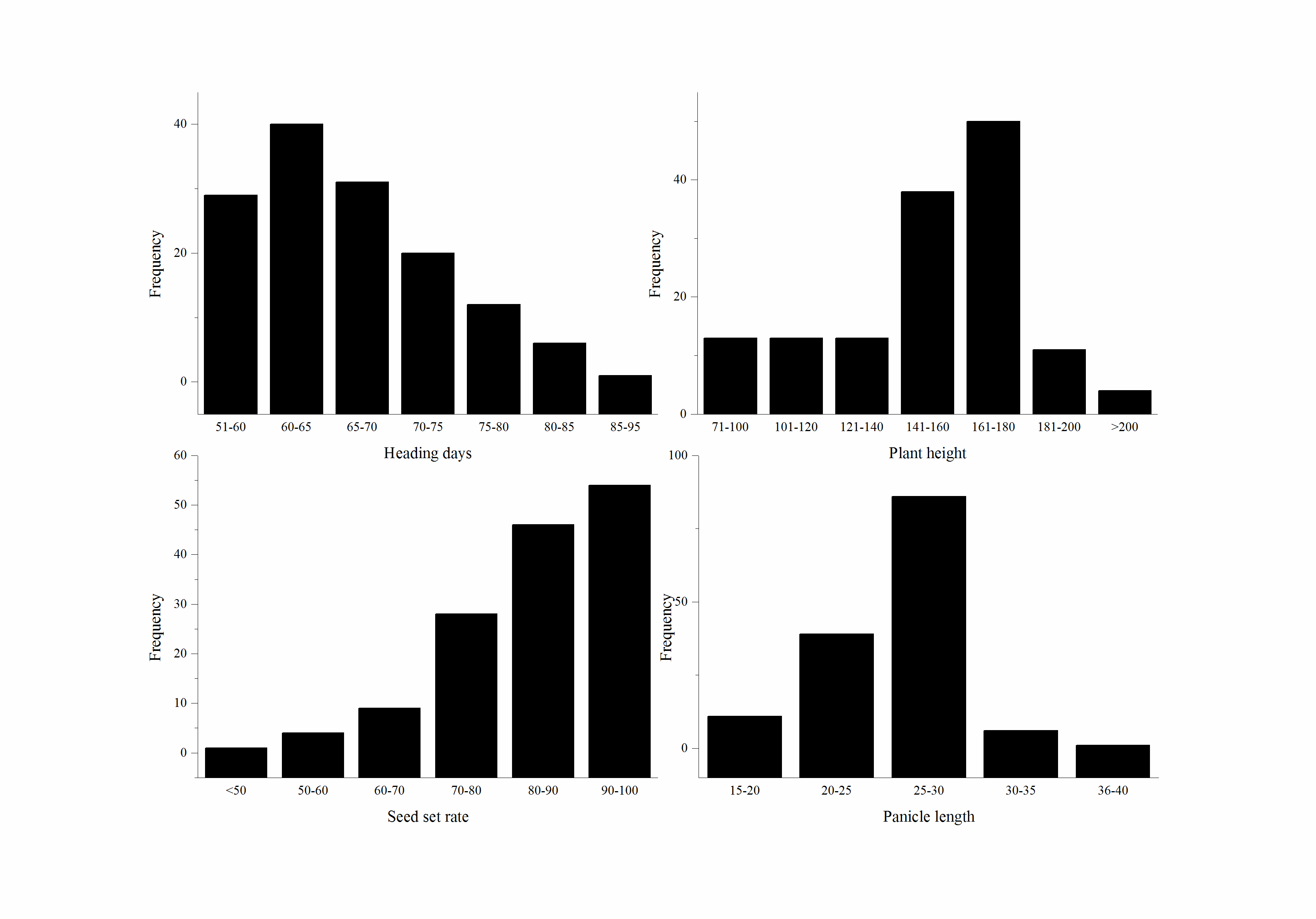


**Figure S4. Frequency distribution of heading days, plant height, seed set rate and panicle length in Panel 1 in 2009.** The height of black bar represents the number of varieties in different range of traits.


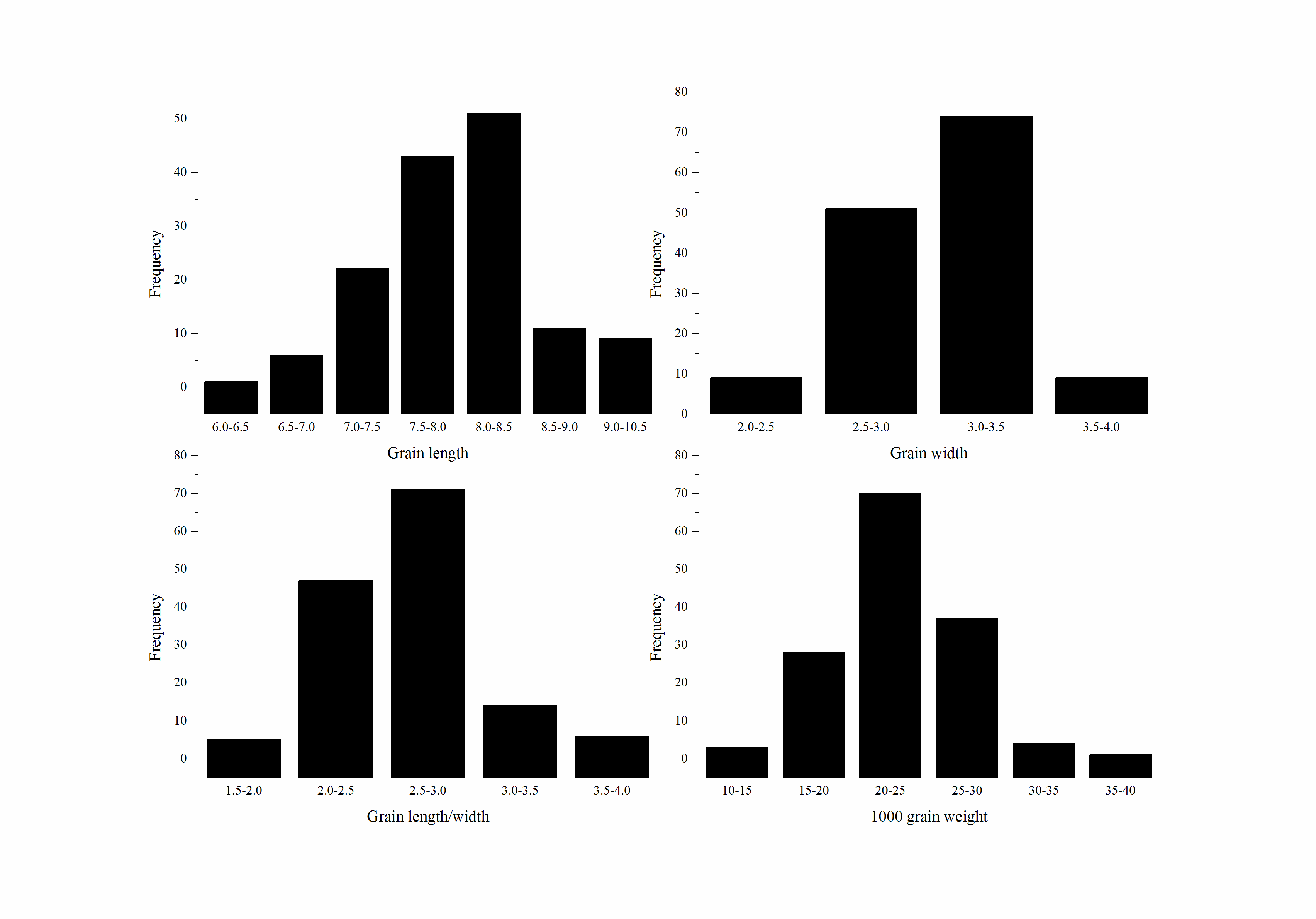


**Figure S5. Frequency distribution of grain length, grain width, grain length/width and 1000 grain weight in Panel 1 in 2009.** The height of black bar represents the number of varieties in different range of traits.


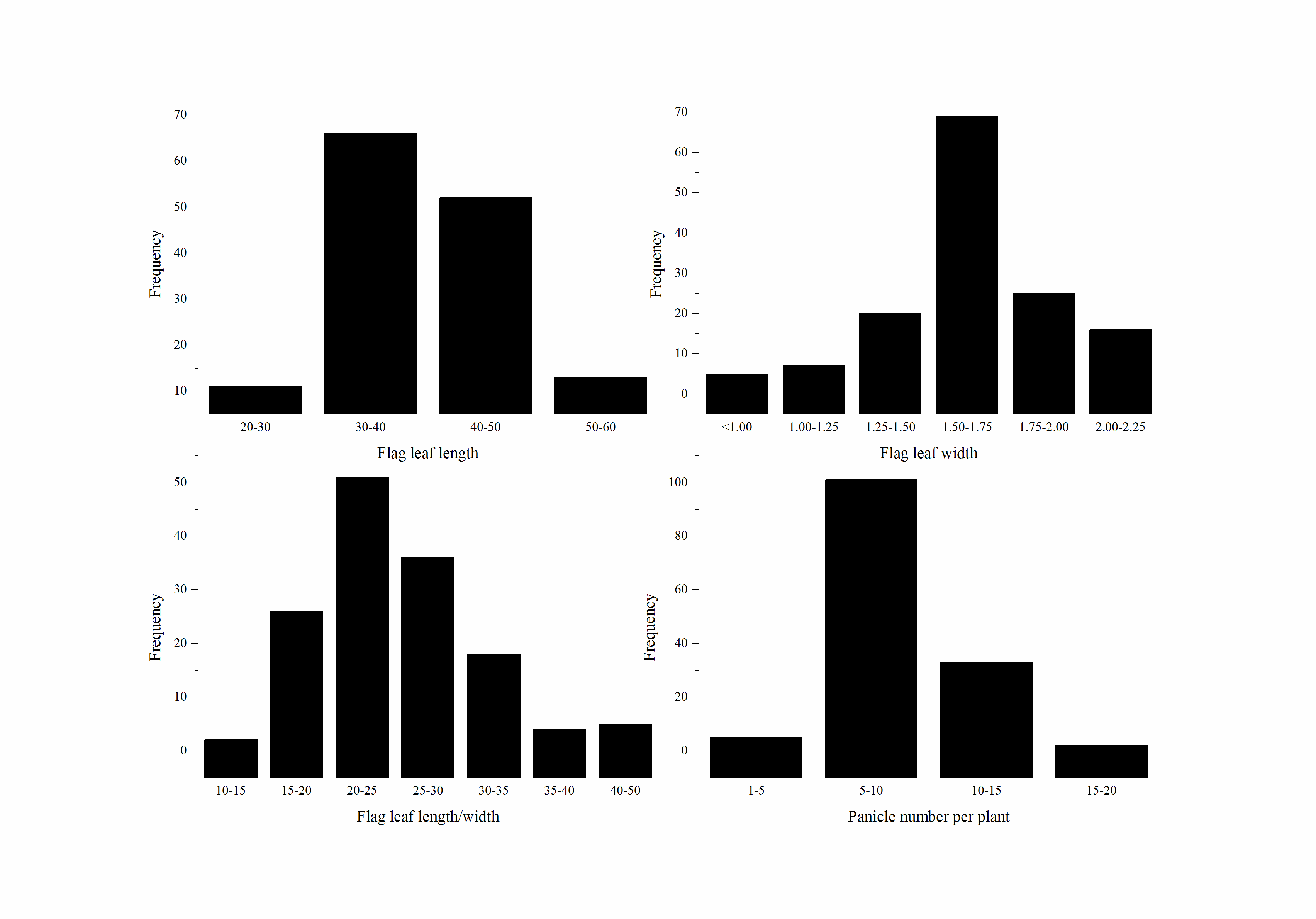


**Figure S6. Frequency distribution of flag leaf length, flag leaf width, flag leaf length/width and panicle number per plant in Panel 1 in 2009.** The height of black bar represents the number of varieties in different range of traits.


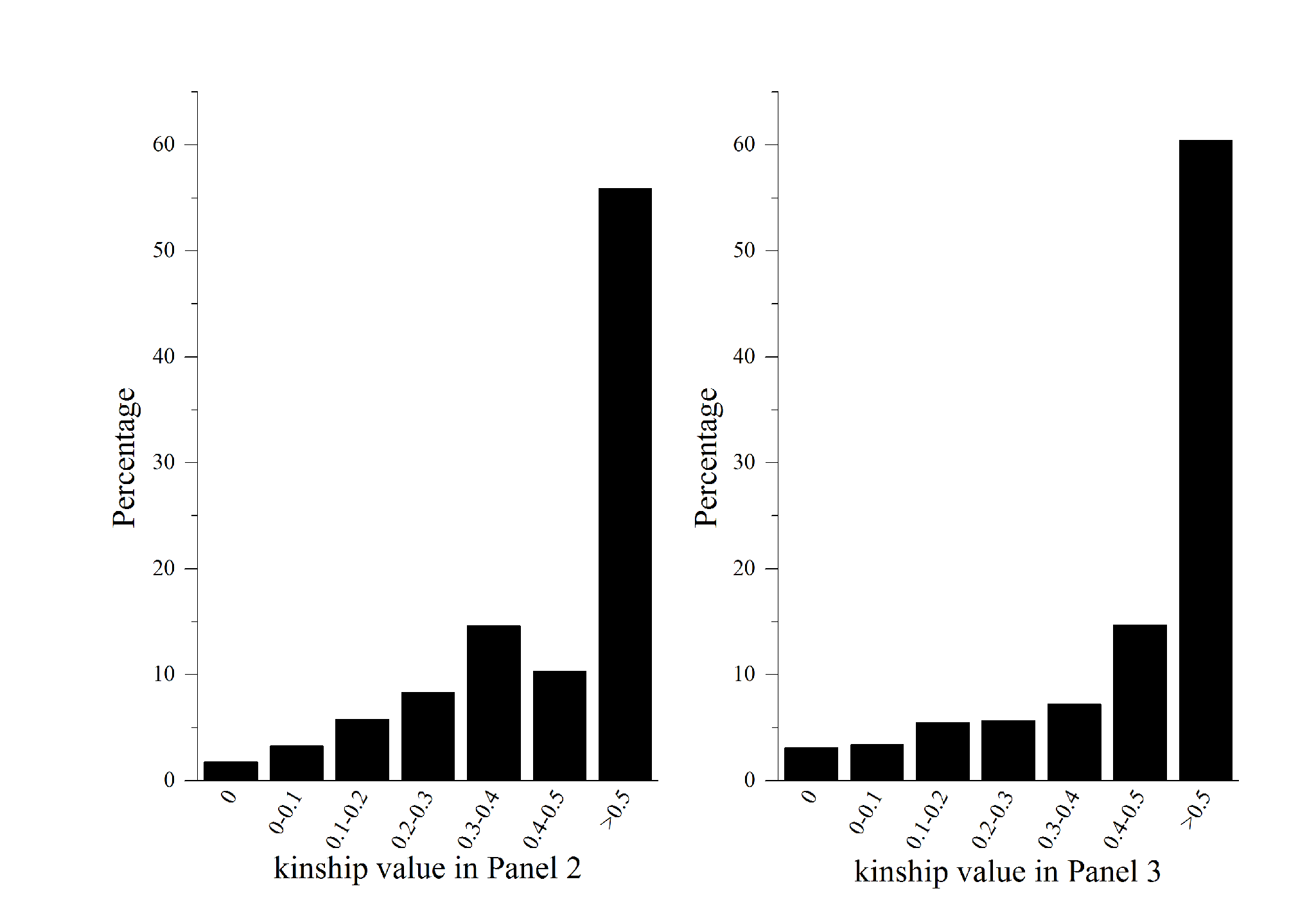


**Figure S7. Distribution of pairwise relative kinship values in Panel 2 and 3.** The height of black bar represents the percentage of varieties in different range of kinships.


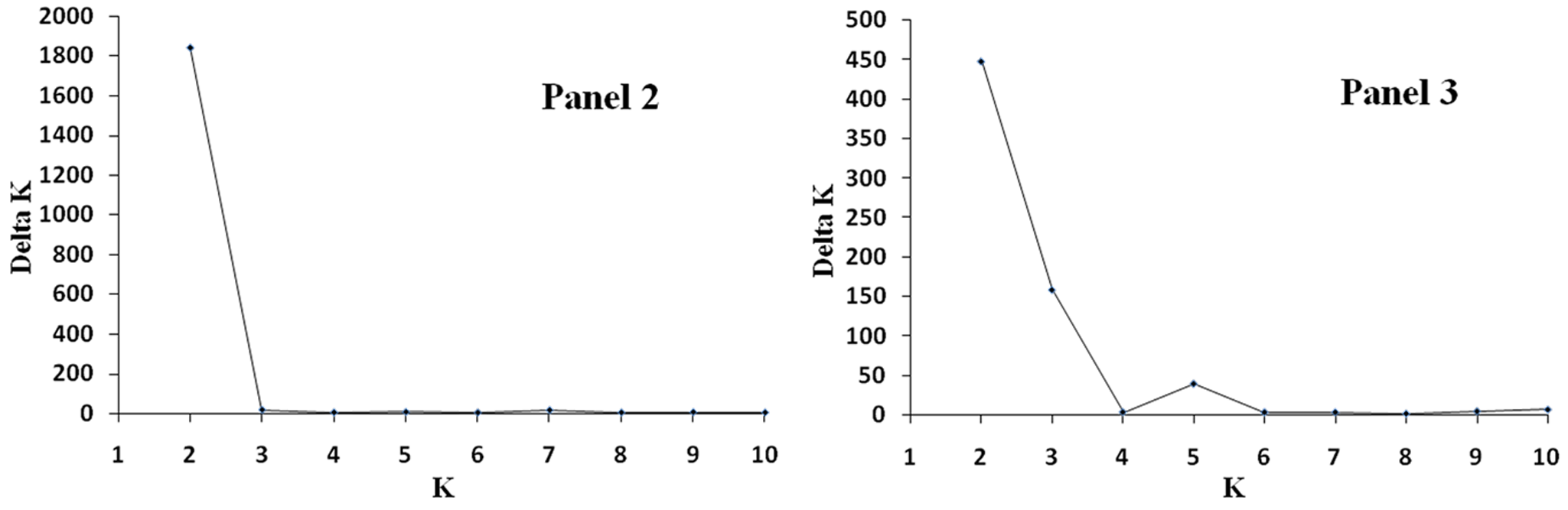


**Figure S8. Delta K change according to different K among Panel 2 and Panel 3 identified by STRUCTURE under Admixture model.**
